# Based on single-cell and transcriptome analysis of inflammatory pathway biomarkers and their molecular mechanisms in chronic obstructive pulmonary disease

**DOI:** 10.1371/journal.pone.0343798

**Published:** 2026-02-25

**Authors:** Yaping Zhou, Hui Gong, Zelin Hao, Lu Wang, Li Li, Xiaoguang Zou

**Affiliations:** 1 The Affiliated Teaching Hospital of Xinjiang Medical University (Affiliated Cancer Hospital), Urumqi, China; 2 Clinical Research Center of Infectious Diseases (Pulmonary Tuberculosis), First People′s Hospital of Kashi, Kashi, China; 3 Department of Respiratory and Critical Care Medicine, First People′s Hospital of Kashi, Kashi, China; 4 Department of Laboratory Medicine, People’s Hospital of Bayingol Mongolian Autonomous Prefecture, Korla, China; Sichuan University, CHINA

## Abstract

**Background:**

Systemic inflammation in chronic obstructive pulmonary disease (COPD) presents significant therapeutic challenges. Our study employs integrated transcriptomic and single-cell analyses to identify inflammation-related biomarkers and elucidate their pathogenic mechanisms in COPD.

**Methods:**

Training dataset GSE37768, validation dataset GSE239897, and single-cell dataset GSE249584 were retrieved from the GEO database. Inflammation-associated genes were screened from the GeneCards database. Differential expression analysis was employed to identify candidate genes, followed by machine learning approaches and expression validation to pinpoint key genes. Functional characterization of these key genes was conducted through Gene Set Enrichment Analysis (GSEA), immune infiltration profiling, molecular regulatory network construction, drug prediction, and GeneMANIA interaction analysis. Single-cell data analysis elucidated cellular heterogeneity and identified critical cell types. Pseudotime analysis was subsequently performed to investigate the roles of key genes throughout developmental trajectories within these critical cell types.

**Results:**

Twelve candidate genes associated with COPD and inflammation were screened, followed by GO and KEGG enrichment analyses. Subsequently, Least Absolute Shrinkage and Selection Operator (LASSO) regression and Support Vector Machine-Recursive Feature Elimination (SVM-RFE) modeling identified six candidate key genes. Among these, only CXCL12, CXCR4, GGT1, and VWF exhibited consistent expression patterns across both training and validation datasets, establishing them as key genes. Their diagnostic value was further validated by constructing an artificial neural network model. Immune infiltration analysis revealed aberrant basophil abundance in COPD. Single-cell analysis annotated 11 distinct cell types, with macrophages representing the sole cell type demonstrating significant abundance differences between COPD and control groups. Pseudotime trajectory analysis delineated nine differentiation states, wherein CXCR4 expression persisted throughout the cellular differentiation trajectory.

**Conclusions:**

This study identified CXCL12, CXCR4, GGT1, and VWF as key genes in COPD pathogenesis. Macrophages constituted the only cell type exhibiting significant abundance alterations, with CXCR4 demonstrating persistent expression throughout macrophage differentiation trajectories. These findings provide valuable insights and suggest potential directions for developing precision therapeutic strategies for COPD.

## Introduction

Chronic Obstructive Pulmonary Disease (COPD) is a common chronic respiratory disease characterized by persistent airway obstruction, lung tissue destruction, and long-term chronic inflammation, leading to high disability and mortality rates [1,2]. The pathological features of COPD primarily include the destruction of lung parenchyma, abnormal degradation of the extracellular matrix, and sustained inflammatory responses in the alveoli and airways. These pathological changes ultimately result in the destruction of alveolar structures and airway remodeling. A large body of research indicates that the onset and progression of COPD are typically caused by prolonged smoking or exposure to air pollutants, such as tobacco smoke and chemical aerosols. These factors cause continuous damage to the epithelial-endothelial cell barrier in the lungs, triggering a series of immune responses, the release of inflammatory mediators, oxidative stress, and autophagy, all of which contribute to disease progression [3,4].

Initially, the inflammatory response in COPD is confined to the lungs, but as the disease progresses, inflammation can extend to systemic circulation, exacerbating comorbidities in the cardiovascular, metabolic, and endocrine systems. The pathological mechanisms of COPD are complex, involving intricate interactions between immunity, metabolism, and structural remodeling, which further hinder the development of effective therapeutic strategies [5]. Although current treatment methods, such as bronchodilators, mucolytics, and corticosteroids, can alleviate symptoms to some extent, they do not significantly slow the progression of the disease or improve patient prognosis [6–8]. Currently, there is no effective treatment available to slow the progression of COPD, thus highlighting the need for new biomarkers and a deeper understanding of the disease′s pathogenesis. Such research is of significant clinical value in improving early diagnosis, predicting disease progression, and developing personalized treatment strategies for COPD.

Existing research on COPD has primarily focused on pulmonary inflammation and the role of related cytokines. Studies have shown that alveolar macrophages, neutrophils, and T lymphocytes (especially TC1, TH1, and TH17 subsets) play key roles in the immune response in COPD [9,10]. The activation of these cells and the secretion of numerous pro-inflammatory cytokines are the main drivers of the chronic inflammatory response in COPD [11]. Additionally, the progression of COPD is associated with immune dysregulation and autophagic dysfunction, and these cellular and molecular changes result in irreversible lung tissue damage [12]. Therefore, further investigation into the inflammation mechanisms, cellular functional changes, and potential biomarkers associated with COPD has become a crucial topic in current COPD research.

Recent advances in single-cell RNA sequencing (scRNA-seq) have begun to elucidate the cellular complexity of the COPD lung. Pioneering studies have successfully mapped the cellular landscape, revealing notable heterogeneity in immune populations such as macrophages and T cells, as well as alterations in epithelial cell states [13–15]. However, many of these studies have focused on characterizing discrete cell types or profiling specific disease stages. A critical gap remains in systematically integrating transcriptional changes across multiple cellular compartments to identify central, multicellular regulatory networks and key hub biomarkers that drive disease progression. Furthermore, the functional continuum and dynamic plasticity of disease-associated macrophage subpopulations, and their precise relationship with disease severity, are yet to be fully defined.

This study aims to combine transcriptomic and single-cell transcriptomic data to comprehensively explore inflammation-related biomarkers in COPD and analyze the functional characteristics and interactions of key immune cells, thereby revealing the potential pathogenic mechanisms of COPD.

To address the aforementioned gaps, we employed an integrative bioinformatics approach. We performed a comprehensive analysis of bulk transcriptomic datasets from public repositories (e.g., GEO) and innovatively correlated the findings with a large-scale, published scRNA-seq atlas of the COPD lung through deep learning-based mapping. This strategy allows us to: (1) bridge the technical divide between bulk and single-cell data, precisely localizing key gene modules identified from bulk analyses to specific cellular subpopulations; (2) uncover core multicellular gene regulatory networks central to COPD inflammation; and (3) identify and preliminarily validate a set of “hub” genes with coordinated expression across cell types, which may serve as potential diagnostic markers or therapeutic targets.

Innovatively, this study will use single-cell RNA sequencing technology to examine the expression profiles and regulatory networks of COPD-related genes at the cellular level, with the goal of providing a theoretical foundation and practical guidance for early diagnosis, precision therapy, and the development of novel targeted therapies for COPD.

## Materials and methods

### Transcriptome differential gene acquisition

The GSE37768 dataset was downloaded from the Gene Expression Omnibus (GEO) database (https://www.ncbi.nlm.nih.gov/geo/) as the training set, comprising 18 chronic obstructive pulmonary disease (COPD) lung tissue samples and 20 control lung tissue samples. The GSE239897 dataset served as the validation set, containing 37 COPD and 43 control lung tissue samples. The single-cell dataset GSE249584, encompassing 11 COPD and 7 healthy control samples, was also acquired. Inflammation-related genes (IRGs) were obtained by searching the keyword “Inflammation” in the GeneCards database (https://www.genecards.org), filtering for genes with a relevance score ≥8, yielding 287 IRGs. GeneCards integrates genomic, transcriptomic, proteomic, and clinical data from over 150 sources. It assigns each gene a “relevance score” reflecting the strength of evidence linking it to a query term (here, “inflammation”). We applied a stringent threshold (score ≥8) to select genes with well-documented inflammatory roles, enhancing the specificity of downstream analysis.

All datasets used in this study were obtained from the publicly available Gene Expression Omnibus (GEO) database. The original studies that generated these data had obtained appropriate ethical approval and participant consent. Therefore, no additional ethical approval was required for the secondary analysis conducted in this work.

### Candidate gene identification and enrichment analysis

Differentially expressed genes (DEGs) in the training set (GSE37768) were identified using the R package “limma” [16] with a significance threshold of P < 0.05 and |log₂FC| > 0. The “ggplot2” [17] and “ComplexHeatmap” packages [18] were employed to generate visualizations via volcano plots and heatmaps, respectively. Subsequently, the intersection between DEGs and IRGs was determined using the “ggVenn” package to identify candidate genes. Gene Ontology (GO) enrichment analysis and Kyoto Encyclopedia of Genes and Genomes (KEGG) pathway enrichment analysis were performed on these candidate genes using the “clusterProfiler” R package [19]. Results were visualized utilizing the “GOplot” package [20].

### PPI network construction

Candidate genes were submitted to the STRING database (https://string-db.org/) [21], applying an interaction score threshold > 0.15. The resulting protein-protein interaction (PPI) network was visualized using Cytoscape software [22].

### Machine learning and expression validation

Least absolute shrinkage and selection operator (LASSO) regression analysis was perforrrrmed using the “glmnet” R package [23], and a support vector machine-Recursive Feature Elimination (SVM-RFE) model was constructed using the “e1071” R package. The “VennDiagram” package was subsequently employed to identify the intersection of genes selected by both LASSO and SVM-RFE, yielding candidate key genes [24]. The expression levels of these candidate key genes were compared between the COPD training set and validation set using the rank-sum test to identify the definitive key genes.

### ANN construction and GSEA analysis

An artificial neural network (ANN) model was built for the key genes within the training set using the “neuralnet” R package. The “c5.go.v7.4.symbols.gmt” gene set from the Molecular Signatures Database (MSigDB) (https://www.gsea-msigdb.org/gsea/msigdb) served as the reference gene set [25]. Spearman correlation analysis was conducted between each key gene and all other genes using the “psych” R package to obtain correlation coefficients(P < 0.05). These coefficients were then ranked. Gene set enrichment analysis (GSEA) was performed on the key genes using the “clusterProfiler” R package [19].

### Immune infiltration analysis and gene function association

Using the xCell algorithm, the differential abundance of 64 immune cell types infiltrating disease and control samples within the training cohort was assessed. The results were visualized using the R package “ggplot2”. Wilcoxon rank-sum tests were employed to identify immune cell types exhibiting significant differential abundance between disease and control samples (P < 0.05), which were subsequently designated as differentially infiltrating immune cells [26]. Functionally associated genes and implicated biological functions for the key genes were predicted using GeneMANIA (http://www.genemania.org/) [27].

### Construction of molecular regulatory networks and drug prediction

To identify potential regulatory miRNAs, the target genes were analyzed using two widely recognized prediction databases: miRDB (http://mirdb.org/) and TargetScan (https://www.targetscan.org/) [28], while key lncRNAs upstream of the targeting miRNAs were retrieved from the starbase database (https://starbase.sysu.edu.cn/) [29]. The miRNA-mRNA-lncRNA molecular regulatory network for the key genes was constructed using Cytoscape software. Transcription factors (TFs) targeting the key genes were identified using the NetworkAnalyst online tool and subsequently visualized with Cytoscape [30]. Potential therapeutic drugs targeting COPD were predicted using the DGIdb database (https://www.dgidb.org/) [31], and the results were visualized with Cytoscape software.

### Single-cell analysis

Single-cell datasets from GSE249584 were processed using the PercentageFeatureSet function in the R package “Seurat” [32] to filter cells and genes based on the following criteria: (1) removal of cells expressing fewer than 200 genes; (2) removal of genes detected in fewer than 3 cells; and (3) retention of cells with between 200 and 3000 expressed genes. These filtered cells were visualized using violin plots generated with the R package “ggplot2” [33]. The top 2000 highly variable genes (HVGs) were identified based on variance stabilization transformation (VST) using the FindVariableFeatures function (with selection.method = “vst”) in “Seurat” for downstream analysis. All samples within the single-cell dataset were normalized using the ScaleData function in “Seurat”. Principal component analysis (PCA) was then performed on the identified HVGs using the prcomp function (typically employed via “Seurat”‘s internal PCA workflow) and visualized with the ElbowPlot function. Subsequently, cell clustering was refined using the JackStraw permutation test algorithm (P < 0.05) [34]. Uniform manifold approximation and projection (UMAP) was applied for dimensionality reduction and cluster visualization.

### Cell annotation and identification of key cell clusters

Marker genes for each cell cluster within the single-cell dataset GSE249584 were identified using the FindAllMarkers function from the “Seurat” R package (criteria: |log_2_FC| > 1; min.pct > 1; FDR < 0.05) [35]. Cell clusters were annotated by referencing the CellMarker database (http://xteam.xbio.top/CellMarker/) [36]. Annotated clusters were visualized via UMAP plots generated with the “umap” R package [37] and dot plots created using “ggplot2”. The PercentageFeatureSet function was employed to quantify the percentage of key genes expressed per cell cluster [38]. Statistically significant differences in cell proportions between control and COPD groups were assessed using the “rstatix” R package, with results presented as boxplots via “ggplot2”.

### Cell communication and pseudotime analysis

Cell communication networks among key cell clusters were analyzed using the “CellChat” R package [39] and visualized with “patchwork” [40]. Pseudotime trajectory analysis for key cells was performed using the “Monocle2” package [41] through the following steps: (1) conversion from a Seurat object to a cell dataset object via the importCDS function; (2) identification of ordering genes (q val < 0.01) using the differentialGeneTest function; (3) dimensionality reduction and clustering with reduceDimension; (4) inference of differentiation trajectories via orderCells; and (5) visualization of results using “plot_pseudotime_heatmap”.

### Statistical analysis

In the violin and box plots, statistical significance was denoted by **** for P < 0.0001, *** for P < 0.001, ** for P < 0.01, * for P < 0.05, and ns for P ≥ 0.05. Unless otherwise specified, P-values were calculated using the Wilcoxon test.

## Results

### Identification and enrichment analysis of candidate genes

**Fig 1:**
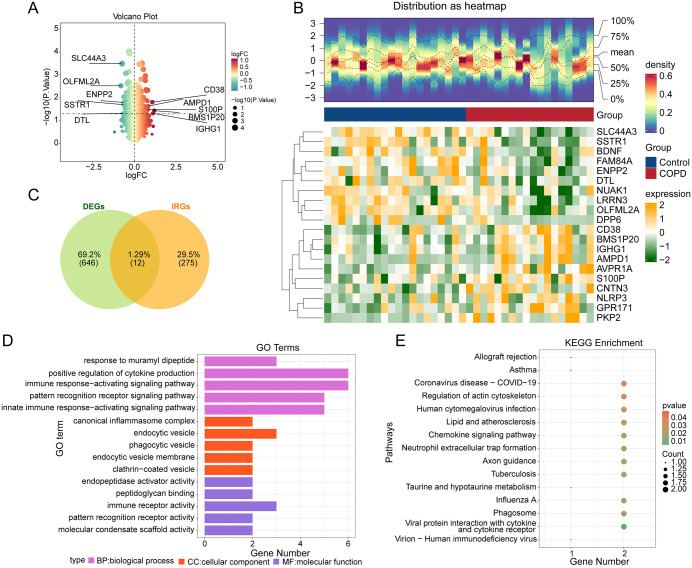
Identification of candidate genes and enrichment analysis. **A** Volcano plot of DEGs in COPD. **B** Heatmap displaying the top 10 upregulated and top 10 downregulated genes ranked by fold change. **C** Venn diagram for screening candidate genes. **D** GO enrichment analysis. **E** KEGG enrichment analysis.

In the training dataset GSE37768, 658 DEGs were identified between the COPD disease group and the normal control group, comprising 263 upregulated genes and 395 downregulated genes (Fig 1A and 1B). To further focus on inflammation-related mechanisms, these DEGs were filtered based on an inflammation relevance score ≥ 8, yielding 12 candidate genes, including NLRP1, HLA-DQB1, VWF, CYBA, CXCR4, NLRP3, CXCL12, GGT1, BDNF, IL17RA, PTPN22, and NOD2 (Fig 1C).

To investigate the potential biological functions and pathways associated with these 12 candidate genes, GO enrichment analysis and KEGG pathway enrichment analysis were performed. The GO analysis yielded 627 significant enrichment results, including 549 biological processes (BC), 20 cellular components (CC), and 58 molecular functions (MF) (Fig 1D). Key significantly enriched GO terms included response to muramyl dipeptide, positive regulation of cytokine production, immune response-activating signaling pathway, pattern recognition receptor signaling pathway, and innate immune response-activating signaling pathway. Furthermore, the 12 candidate genes were significantly enriched in 39 KEGG pathways (Fig 1E), such as Allograft rejection, Asthma, Coronavirus disease - COVID-19, Regulation of actin cytoskeleton, Human cytomegalovirus infection, Lipid and atherosclerosis, Chemokine signaling pathway, Neutrophil extracellular trap formation, Axon guidance, and Tuberculosis. To investigate functional associations between the candidate gene products, a PPI network was constructed for their encoded proteins. This network consisted of 12 nodes connected by 45 interaction edges (S1 Fig).

### Identification of key genes

**Fig 2:**
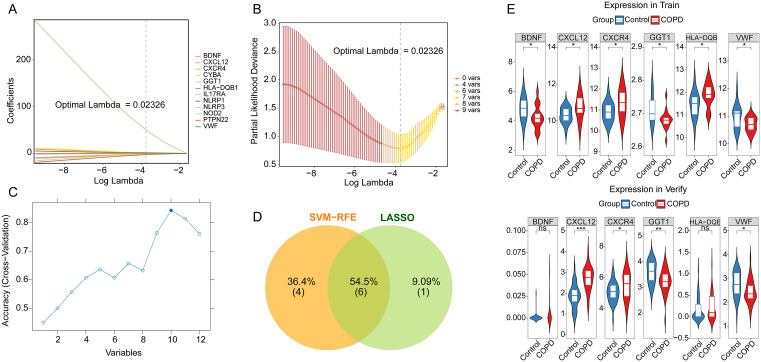
Machine learning and expression validation for identifying key genes. **A** Lasso coefficient path plot for candidate genes. **B** Lasso cross-validation curve. **C** SVM-RFE analysis of candidate genes. **D** Venn diagram identifying candidate key genes. **E** Expression levels of candidate key genes in the training and validation sets.

Using the training dataset, Lasso regression analysis was performed on the 12 candidate genes (Fig 2A and 2B). The optimal regularization strength parameter lambda was determined via cross-validation, screening out 7 candidate genes with non-zero regression coefficients, including HLA-DQB1, VWF, CXCR4, CXCL12, GGT1, BDNF, and NOD2. At this lambda value, the model achieved optimal predictive performance on the training dataset. The importance of the candidate genes was further evaluated using an SVM-RFE model (Fig 2C). Model prediction accuracy peaked when the number of feature genes was 10, which consisted of HLA-DQB1, PTPN22, CYBA, CXCL12, CXCR4, VWF, GGT1, NLRP1, BDNF, and NLRP3. The intersection of the genes identified by these two methods yielded 6 candidate key genes, namely HLA-DQB1, VWF, CXCR4, CXCL12, GGT1, and BDNF (Fig 2D). Among these 6 candidate key genes, only CXCL12, CXCR4, GGT1, and VWF exhibited consistent expression trends between the training and validation sets, with CXCL12 and CXCR4 upregulated and GGT1 and VWF downregulated (Fig 2E). Consequently, CXCL12, CXCR4, GGT1, and VWF were designated as key genes.

### Construction of an artificial neural network diagnostic model

Based on the identified key genes (CXCL12, CXCR4, GGT1, VWF), an ANN classification model was constructed to distinguish COPD patients from healthy controls. The multilayer perceptron (MLP) classifier comprised one hidden layer containing three neurons (S2A Fig). While the model’s initial performance metrics appeared high, we acknowledge that further validation is required in larger and independent cohorts to confirm its generalizability. Within the current analytical framework, we observed that the positive weight value between neuron H1 and output neuron O2 (representing COPD) was the largest positive weight in the network, suggesting that activation of the H1 node may contribute to distinguishing the COPD class. Conversely, the negative weight value between H1 and output neuron O1 (representing healthy control, CK) was the largest negative weight in absolute magnitude, indicating that activation of the H1 node may exert an inhibitory effect on predicting the normal class. These weight patterns provide preliminary insights into how the model processes the input gene features, though their biological interpretation requires further investigation. Subsequently, confusion matrices generated for both the training and validation sets demonstrated that all COPD patient samples and all healthy control samples were correctly classified (S2B Fig).

### GSEA enrichment analysis of key genes

**Fig 3:**
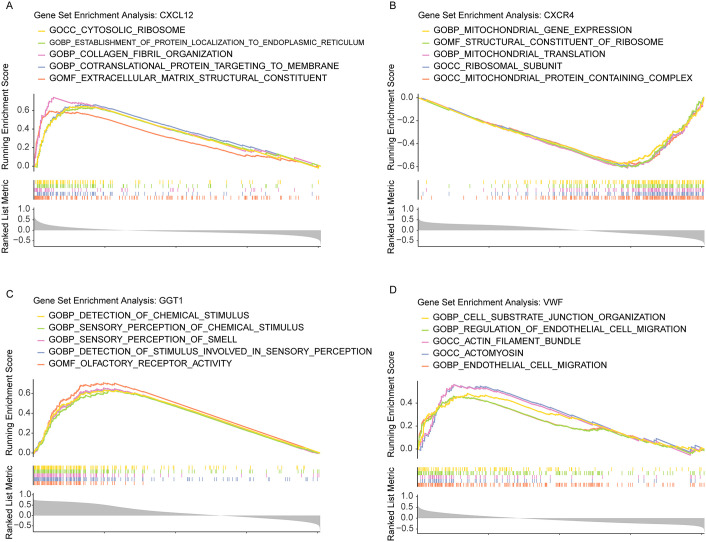
GSEA enrichment analysis of key genes. **A** CXCL12 **B** CXCR4 **C** GGT1 **D** VWF.

GSEA was performed on the key genes (CXCL12, CXCR4, GGT1, VWF) to elucidate their associated biological functions (Fig 3). CXCL12 showed significant enrichment in functions including CYTOSOLIC RIBOSOME, ESTABLISHMENT OF PROTEIN LOCALIZATION TO ENDOPLASMIC RETICULUM, COLLAGEN FIBRIL ORGANIZATION, COTRANSLATIONAL PROTEIN TARGETING TO MEMBRANE, and EXTRACELLULAR MATRIX STRUCTURAL CONSTITUENT. CXCR4 was significantly enriched in MITOCHONDRIAL GENE EXPRESSION, STRUCTURAL CONSTITUENT OF RIBOSOME, MITOCHONDRIAL TRANSLATION, RIBOSOMAL SUBUNIT, and MITOCHONDRIAL PROTEIN-CONTAINING COMPLEX. GGT1 exhibited significant enrichment in DETECTION OF CHEMICAL STIMULUS, SENSORY PERCEPTION OF CHEMICAL STIMULUS, SENSORY PERCEPTION OF SMELL, DETECTION OF STIMULUS INVOLVED IN SENSORY PERCEPTION, and OLFACTORY RECEPTOR ACTIVITY. VWF demonstrated significant enrichment in CELL-SUBSTRATE JUNCTION ORGANIZATION, REGULATION OF ENDOTHELIAL CELL MIGRATION, ACTIN FILAMENT BUNDLE, ACTOMYOSIN, and ENDOTHELIAL CELL MIGRATION.

### Immune cell infiltration analysis

**Fig 4:**
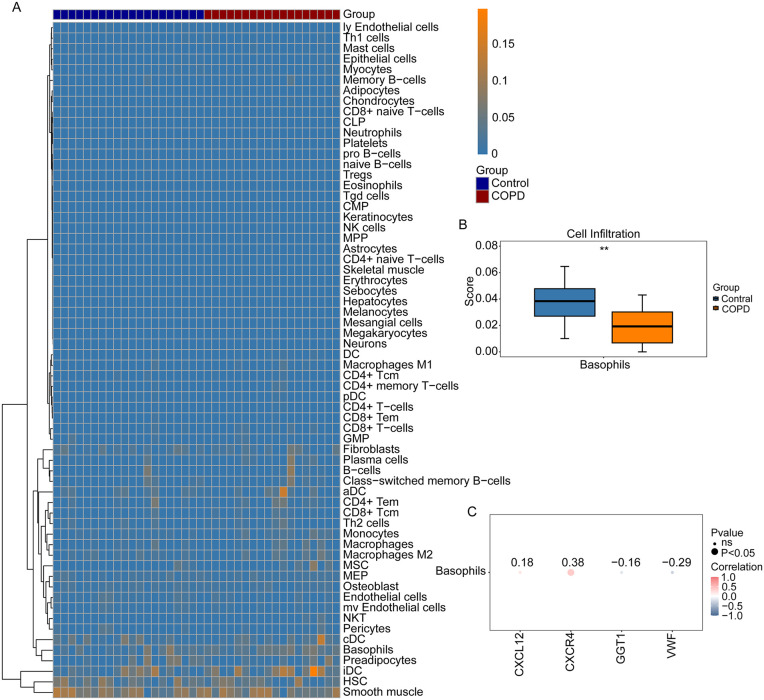
Immune cell infiltration analysis. **A** Heatmap of immune cell enrichment scores for COPD and control group samples. **B** Box plot of the significantly different immune cell (Basophils) in COPD and control group samples. **C** Correlation analysis between key genes and the differential immune cell.

To explore immune infiltration in COPD, the xCell algorithm was employed to investigate the infiltration abundance of 64 immune cell types across all samples in the training set. Based on sample grouping, the Wilcoxon rank-sum test identified Basophils as the sole immune cell exhibiting a statistically significant difference (P < 0.01) between disease and control samples, designated as the differential immune cell (Fig 4A and 4B). Basophils exhibited the highest correlation with CXCR4 among the key genes (Fig 4C).

### GeneMANIA analysis of hub genes and construction of the molecular regulatory network

Co-expression networks for the hub genes were constructed to elucidate gene-gene interactions (S3 Fig). Significant functional enrichment was observed for processes including cellular response to chemokine, cytokine activity, leukocyte chemotaxis, neutrophil migration, G protein-coupled receptor binding, fatty acid derivative metabolic process, and blood coagulation.TargetScan predicted 16 miRNAs, while miRDB predicted 74 miRNAs. The union set of predicted miRNAs from both databases, after deduplication, yielded 85 unique miRNAs for subsequent identification of upstream lncRNAs. Based on this miRNA set, 90 candidate lncRNAs predicted to regulate them upstream were identified. Subsequently, an miRNA-mRNA-lncRNA molecular regulatory network for the hub genes was constructed (S4A Fig). To identify TFs potentially directly regulating the expression of the hub genes, the NetworkAnalyst online tool was employed. This analysis predicted 9 TFs for CXCL12, 16 TFs for CXCR4, 4 TFs for VWF, and 15 TFs for GGT1 (S4B Fig).

### Drug prediction

Potential targeted drugs interacting with the hub genes were queried in the DGIdb database. This identified 25 potentially interacting drugs for CXCR4, 8 for CXCL12, 20 for VWF, and 27 for GGT1 (S5 Fig), providing valuable leads for exploring therapeutic strategies targeting these genes.

### Single-Cell Analysis

**Fig 5:**
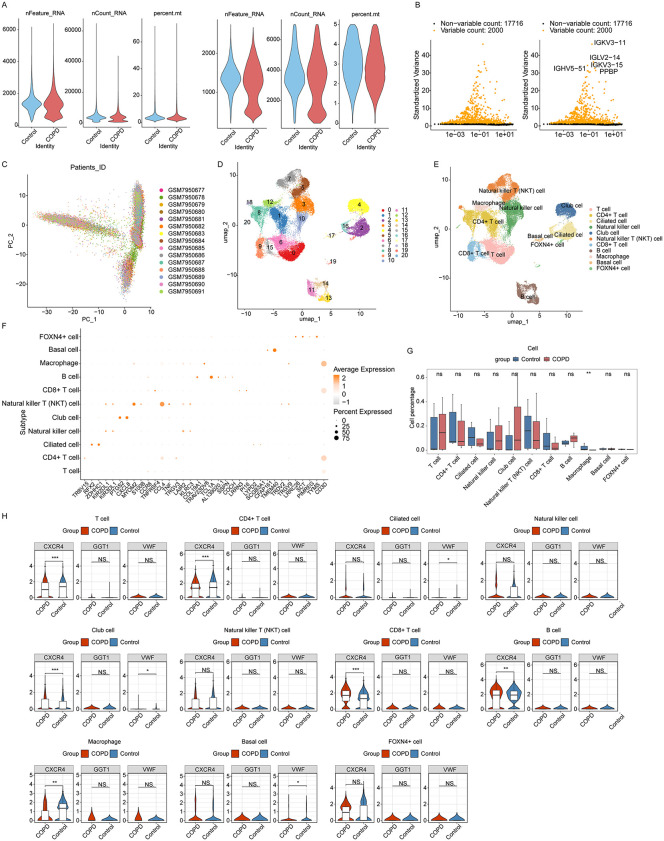
Single-cell analysis. **A** Distribution plots of nFeature_RNA, nCount_RNA, and percent.mt pre- and post-quality control (left: pre-QC, right: post-QC). **B** Highly variable gene selection. **C** PCA dimensionality reduction. **D** UMAP clustering plot. **E** UMAP plot annotated with cell types. **F** Expression of marker genes within annotated cell types. **G** Box plots comparing the abundance of annotated cell types between Control and COPD groups. **H** Expression of key genes within annotated cell types.

Rigorous quality control was first implemented to enhance data reliability, followed by normalization to stabilize variance and reduce interference from less relevant genes (Fig 5A). The top 2,000 highly variable genes were selected for subsequent PCA dimensionality reduction (Fig 5B). The top 30 statistically significant principal components from the PCA were chosen for clustering analysis. UMAP was used to visualize the dimensionality reduction results, yielding 21 distinct cell clusters (Fig 5C, 5D and S6). Cell clusters were annotated by cross-referencing cluster-specific marker genes with classical marker genes for relevant cell types obtained from the CellMarker database. This process annotated 11 cell types (Fig 5E), including Natural killer T (NKT) cell, CD8 + T cell, B cell, Macrophage, Basal cell, T cell, CD4 + T cell, Ciliated cell, Natural killer cell, Club cell, and FOXN4 + cell.

Key genes were projected onto the annotated cells to determine their expression patterns within each cell type. Key genes were primarily expressed in 8 cell types, including CD8 + T cell, B cell, Macrophage, Basal cell, T cell, CD4 + T cell, Ciliated cell and Club cell, with CXCL12 exhibiting no detectable expression in any annotated cell type. Strikingly, among the 11 annotated cell types, only Macrophages showed a significant difference between the control and COPD groups (Fig 5F-H). The proportion of macrophages showed a significant increase in the COPD group (rising from 34.6% to 52.4%), while the proportions of natural killer cells (decreasing from 17.3% to 8.5%) and T cells (decreasing from 23.7% to 16.8%) were markedly reduced. The high expression of key genes within Macrophages, coupled with their significant inter-group difference, suggested a pivotal role for Macrophages in COPD pathogenesis. Consequently, Macrophages were identified as the key cell cluster for subsequent analysis.

### Communication analysis and pseudotime analysis of the key cell cluster

**Fig 6:**
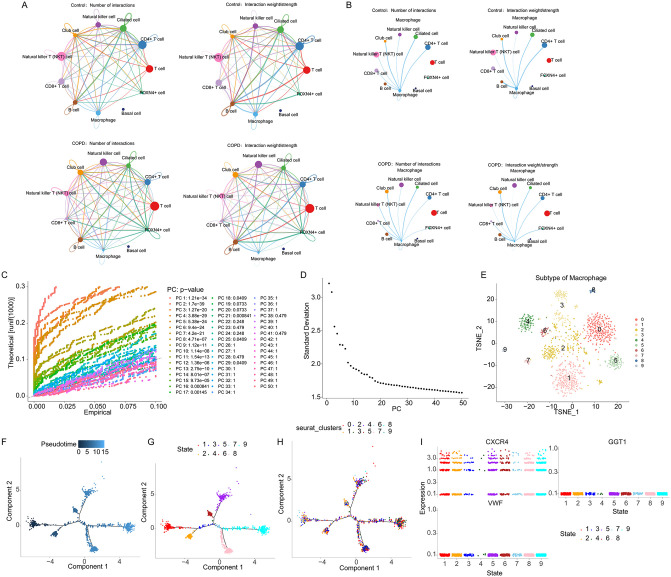
Communication analysis and pseudotime analysis of the key cell cluster. **A** Cell-cell communication interaction network. **B** Macrophage-specific communication interaction network. **C** Dimensionality reduction plot of the key cell cluster. **D** PCA elbow plot. **E** Re-dimensionality reduction plot of the key cell cluster. **F** Pseudotime analysis of the key cell cluster. **G** Differentiation states along the trajectory. **H** Trajectory plot of cell subclusters. **I** Expression dynamics of key genes during the inferred cellular differentiation.

Leveraging the annotated 11 cell clusters, a cell-cell communication network was constructed using CellChat (Fig 6A). Within the disease group, ligand-receptor interactions were identified between Macrophages and all other cell clusters except T cells and Basal cells (Fig 6B). To dissect the intrinsic heterogeneity of macrophages and their potential functional subtypes in COPD, macrophages were subjected to re-dimensionality reduction and subclustering, yielding 10 distinct macrophage subclusters (Fig 6C-E). Subsequently, to explore potential state transitions and differentiation trajectories among macrophage subclusters, pseudotime analysis was performed based on the annotated subclusters (Fig 6F). The cellular trajectory was segmented according to trajectory nodes, revealing 9 distinct transition states (Fig 6G). Projecting the subclusters back onto the pseudotemporal trajectory confirmed alignment with their original annotated states (Fig 6H). Notably, CXCR4 demonstrated dynamic expression throughout the inferred differentiation process. While this observation is intriguing and suggests a potential role in macrophage differentiation, it remains correlative. Future experimental studies are required to establish whether CXCR4 plays a causal role in COPD pathogenesis(Fig 6I).

## Discussion

COPD is a common chronic respiratory disease typically triggered by long-term smoking or exposure to air pollutants, presenting with persistent airflow limitation and progressive lung function decline [42]. With the global aging population and increasingly severe air pollution issues, COPD has become one of the leading causes of morbidity and mortality worldwide [43,44]. Despite progress in understanding the pathogenesis, clinical features, and treatment methods of COPD in recent years, the complexity of its pathological mechanisms and clinical heterogeneity still pose significant challenges to early diagnosis and treatment [45]. In particular, clinical diagnosis currently relies primarily on pulmonary function tests and symptom assessments, which are often ineffective in the early stages of the disease, highlighting the urgent need for the development of new biomarkers and early diagnostic tools. This study integrated multi-omics data analysis to explore key inflammatory pathways and identify potential biomarkers, thereby providing new insights into the molecular mechanisms of COPD.

### Key genes and pathways

Traditional research on the molecular mechanisms of COPD mainly relies on DEG screening, yet this method often overlooks the complex interactions between genes and their dynamic changes during disease progression. Therefore, we propose an innovative “inflammation-related score-guided multi-level screening strategy”, which utilizes a quantitative inflammation response score (≥8 threshold) to systematically screen 658 DEGs and accurately capture 12 core IRGs. This strategy not only improves the targeting of key biomarkers but also avoids the selection bias inherent in traditional methods, ensuring the biological relevance of the identified targets.

Compared to traditional GSEA-based methods, this study further validated the selected core genes (CXCL12, CXCR4, GGT1, VWF) using machine learning models (LASSO regression and SVM-RFE) [46] It is important to note that the involvement of the CXCR4/CXCL12 signaling axis in COPD has been documented in previous studies. The novelty of our work lies not in its initial discovery, but in the systematic validation of its central role through our integrated multi-omics and machine learning framework, and in the single-cell resolution mapping of CXCR4 expression dynamics within macrophage subpopulations. Similarly, VWF has been reported as a marker of endothelial dysfunction in COPD and other inflammatory disorders. Our study reinforces its significance by linking it to vascular remodeling pathways (e.g., “endothelial cell migration”) through GSEA and positioning it within a core molecular network alongside immune-related genes like CXCR4, suggesting potential crosstalk between immune dysregulation and vascular injury in COPD. A critical comparison with prior large-scale transcriptomic studies (e.g., GSE47460, GSE76925) confirms the recurrence of these pathways while highlighting our contribution in refining their context through advanced analytical integration.

### Macrophage Heterogeneity and Immune Interactions

Single-cell transcriptomics data provided robust support for this study. Single-cell transcriptomics analysis revealed that macrophages were the immune cells showing significant differences in abundance between the COPD and control groups.

In our single-cell clustering analysis, the cluster annotated as “-FOXN4+ cells”- likely represents a population of basal or epithelial progenitor cells in lung tissue. This inference is based on the expression of canonical basal cell markers (e.g., KRT5, TP63) alongside FOXN4, which has been implicated in epithelial development and repair. However, given the relatively low frequency and unusual designation of this cluster, we explicitly acknowledge in the Limitations section that further validation—for instance, using spatial transcriptomics or immunohistochemistry on lung tissue sections—is necessary to confirm its identity and functional relevance in COPD.

More importantly, the high expression of CXCR4 in macrophages suggests its potential key role in the immune response of COPD. Single-cell subgroup analysis further revealed that CXCR4 continuously expresses during macrophage differentiation and its expression spans across 9 macrophage subpopulations. This finding challenges the traditional view of “neutrophils dominating COPD inflammation” and offers a new understanding of the complexity of COPD’s immune response [47].

### Potential clinical implications and future directions

To better apply these molecular biomarkers for clinical diagnosis, this study constructed an artificial neural network diagnostic model based on the selected key genes (CXCL12, CXCR4, GGT1, VWF). While the model demonstrated strong discriminatory power within our dataset, we emphasize that its performance requires further validation in larger, independent prospective cohorts. The model’s internal weight analysis suggested that specific nodes (e.g., neuron H1) were highly influential in classification, hinting at a potential synergistic effect among the key genes. [48].

In drug prediction, the study systematically screened and identified 20 potential targeted drugs, which may provide valuable clues for the combined treatment of COPD and comorbid cardiovascular complications [49]. For instance, some drugs targeting the CXCL12/CXCR4 axis or VWF pathway may play important roles in alleviating COPD-related inflammation and vascular remodeling. Future studies can validate the efficacy of these drugs through clinical trials and explore their potential in early COPD intervention.

This study, through the integration of multi-omics data, has provided an in-depth understanding of the molecular mechanisms of COPD and potential therapeutic targets. By precisely screening key genes, constructing efficient diagnostic models, and analyzing the immune microenvironment, especially the heterogeneity of macrophages, we propose a new mechanism of immune imbalance in COPD. Our research not only expands the understanding of the CXCL12/CXCR4 axis in COPD but also provides new insights into the role of VWF in vascular remodeling. We note that xCell may introduce algorithmic biases when estimating certain immune cell subtypes (e.g., alveolar macrophages, neutrophils) due to overlapping gene signatures and tissue-specific expression backgrounds. To enhance robustness, we have repeated the immune infiltration analysis using CIBERSORTx and included comparative results in the supplementary materials.

Although this study has thoroughly analyzed COPD from the perspectives of molecular mechanisms, immune cell infiltration, and machine learning models, many areas remain to be explored. Future research should focus on the spatial heterogeneity of the immune microenvironment, the role of metabolic reprogramming, dynamic monitoring of key genes, and the development of personalized diagnostic models. These studies will not only deepen our understanding of the molecular mechanisms of COPD but also provide a theoretical foundation and experimental basis for early diagnosis, precision treatment, and the development of personalized treatment strategies.

### Study limitations

This study has several limitations that should be acknowledged. First, our analyses are based entirely on publicly available genomic datasets. While this allows for comprehensive bioinformatics exploration, it necessitates cautious interpretation and requires experimental validation in wet-lab settings. Second, the heterogeneity across different datasets (e.g., sample sources, platforms) could introduce biases, despite our efforts to identify consensus signals. Third, for the scRNA-seq analysis, the relatively small cohort size (18 samples) may limit the statistical power to detect subtle differences in rare cell subtypes. Additionally, although we applied batch correction, potential residual technical variation between samples could influence the clustering and interpretation. Fourth, our conclusion that macrophages were the primary cell type showing a significant abundance difference, while robust within the scope of our analysis, should be interpreted with the recognition that deeper profiling of T-cell and epithelial subpopulations might reveal more nuanced, state-specific alterations. Fifth, the study design is cross-sectional, which limits our ability to infer causal relationships between gene expression changes and disease progression. Finally, the immune cell infiltration results from bulk RNA-seq, particularly regarding rare cell types like basophils, are derived from computational deconvolution and should be confirmed with orthogonal methods such as flow cytometry or spatial transcriptomics. Addressing these limitations will be essential in future work to translate these computational insights into clinical applications.

## Conclusion

This study systematically explored the molecular landscape of COPD through integrated multi-omics analysis. By leveraging inflammation-related gene sets and machine learning approaches, we identified CXCL12, CXCR4, GGT1, and VWF as key inflammatory biomarkers associated with COPD. Our single-cell analysis further highlighted the central role of macrophages and the persistent expression of CXCR4 during macrophage differentiation, suggesting its potential involvement in COPD pathogenesis. These findings provide valuable hypotheses and candidate targets for future experimental validation and may contribute to a deeper understanding of COPD mechanisms.

## Supporting information

S1 Fig Protein interaction network. (TIF)

S2 Fig Artificial neural network and confusion matrix results. (TIF)

S3 Fig Key gene co-expression network. (TIF)

S4 Fig Molecular regulatory networks and transcription factors regulating the expression of key genes. (TIF)

S5 Fig Potential targeted drugs. (TIFF)

S6 Fig Principal component cluster analysis. (TIF)

S7 Fig Flow chart of analysis. (TIF)

S1 Table Evaluate model performance. (XLSX)

S2 Table Summary of Key Inflammatory Biomarkers Identified in Chronic Obstructive Pulmonary Disease. (XLSX)

S3 Table Dataset Characteristics and Preprocessing. (XLSX)

S4 Table A complete table of marker genes. (XLSX)

S1 File ANN – Performance and specificity of the model. ssGSEA-Validation Set – Immune infiltration analysis of validation set. rstudio-export – Differences in cell number and proportion.
(ZIP)

S2 File rstudio-export. (ZIP)

S3 File ssGSEA-Validation Set. (ZIP)

## Abbreviations

COPD: Chronic obstructive pulmonary disease
GSEA: Gene Set Enrichment Analysis
GEO: Gene Expression Omnibus
IRGs: Inflammation-related genes
DEGs: Differentially expressed genes
GO: Gene Ontology
KEGG: Kyoto Encyclopedia of Genes and Genomes
PPI: Protein-protein interaction.
