# Supplementary figures and images for: Based on single-cell and transcriptome analysis of inflammatory pathway biomarkers and their molecular mechanisms in chronic obstructive pulmonary disease
Source: PLoS One. 2026 Feb 25;21(2):e0343798. doi: 10.1371/journal.pone.0343798 (PMC12935203; doi:10.1371/journal.pone.0343798)

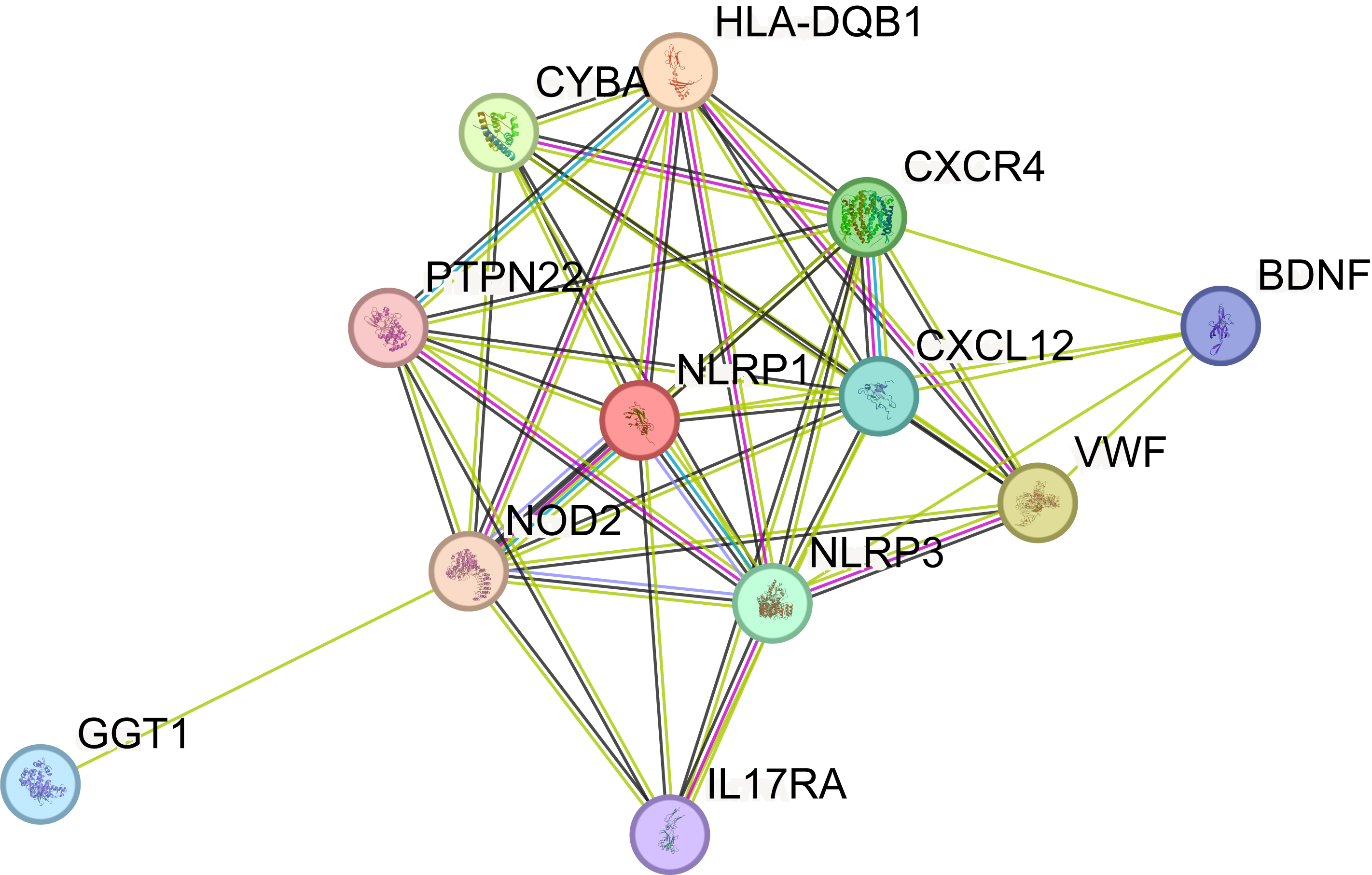

Supplement: S1 Fig — (TIF) [file pone.0343798.s001.tif]

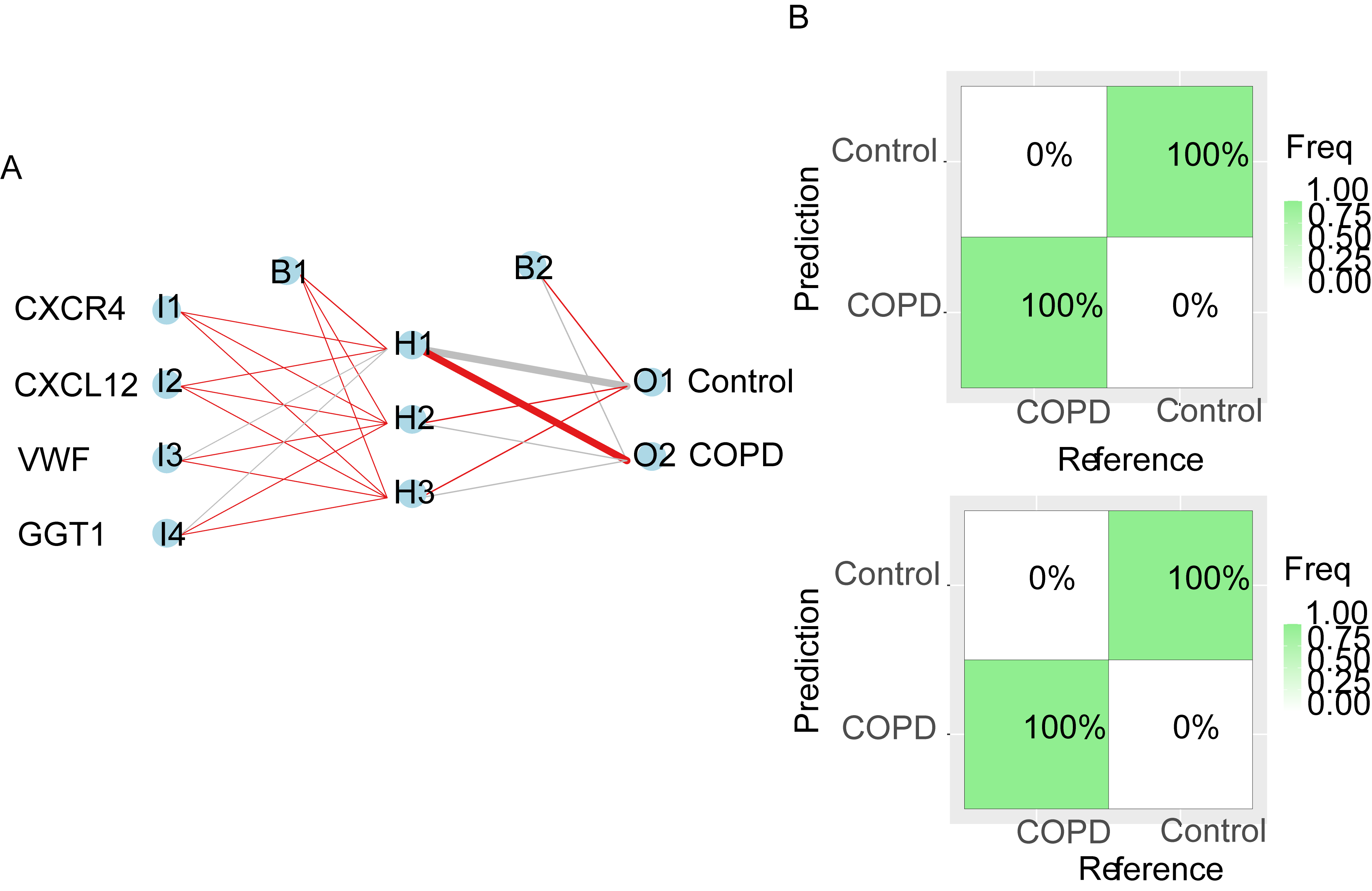

Supplement: S2 Fig — (TIF) [file pone.0343798.s002.tif]

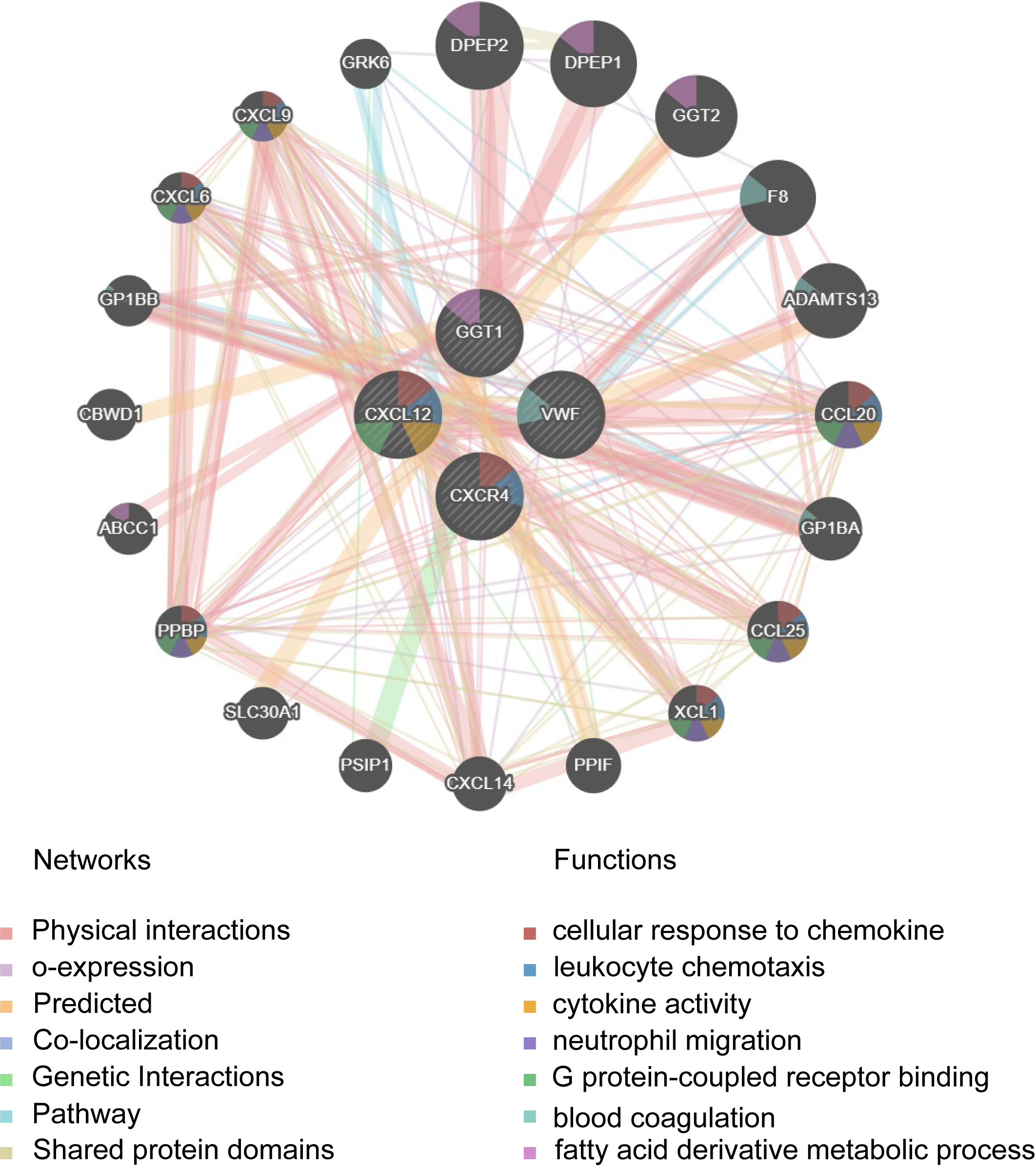

Supplement: S3 Fig — (TIF) [file pone.0343798.s003.tif]

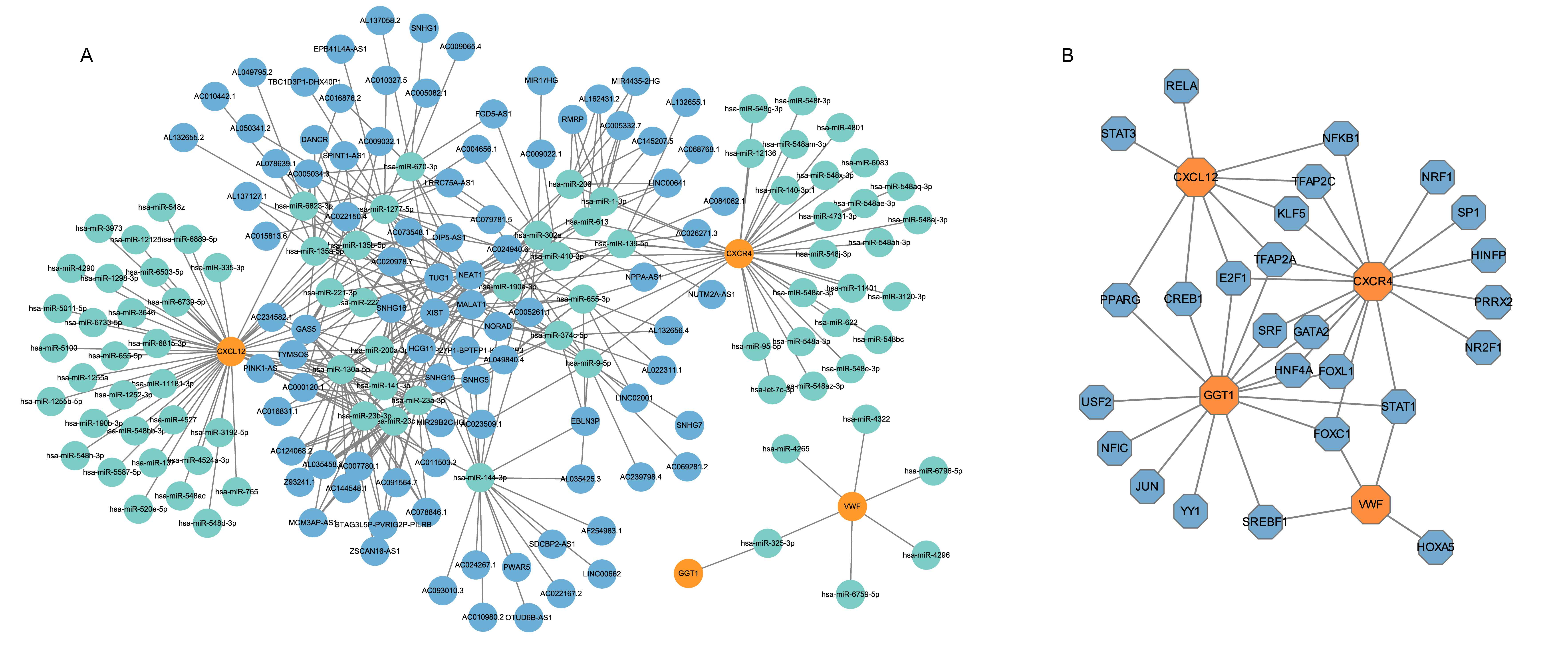

Supplement: S4 Fig — (TIF) [file pone.0343798.s004.tif]

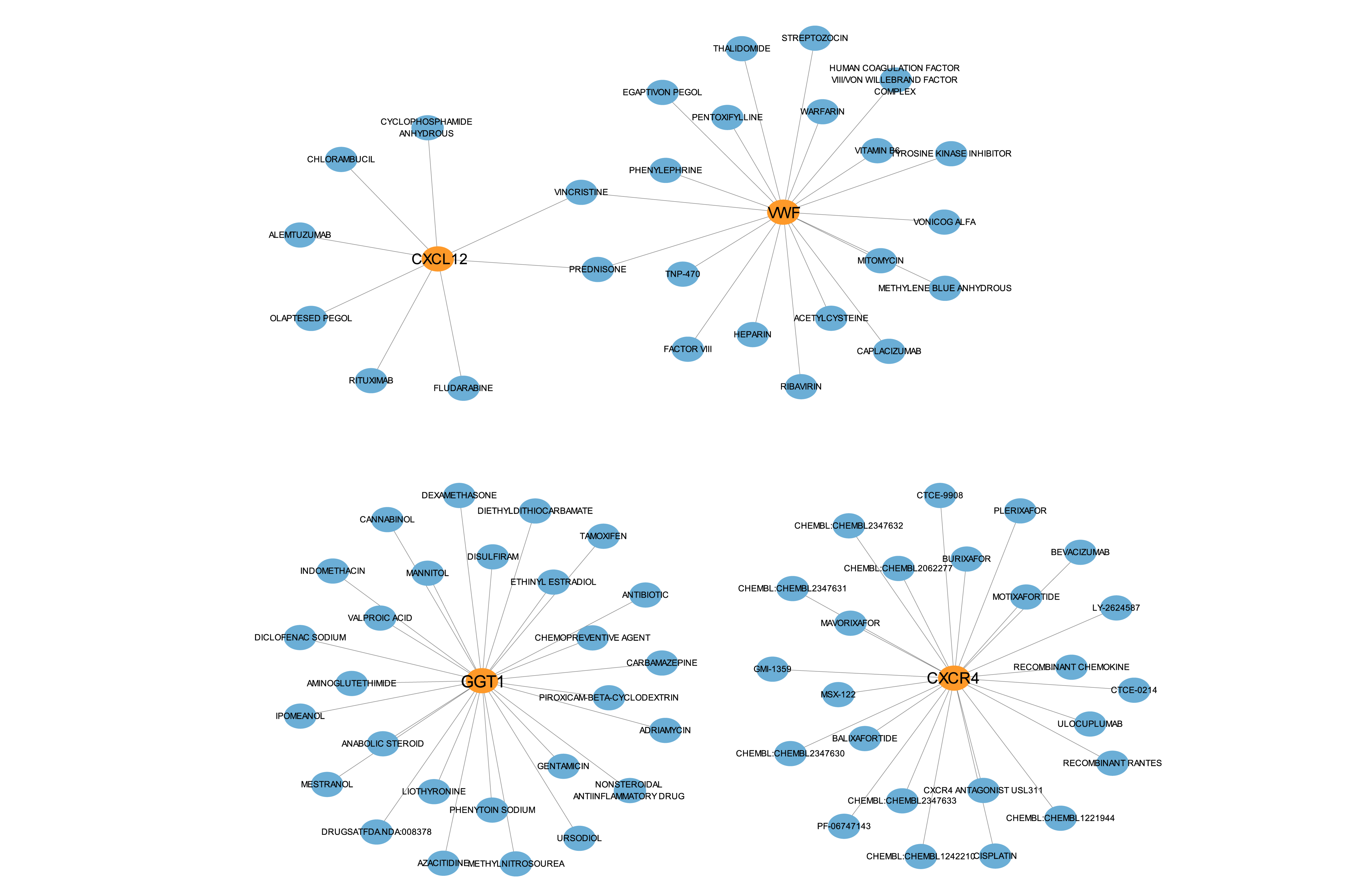

Supplement: S5 Fig — (TIFF) [file pone.0343798.s005.tiff]

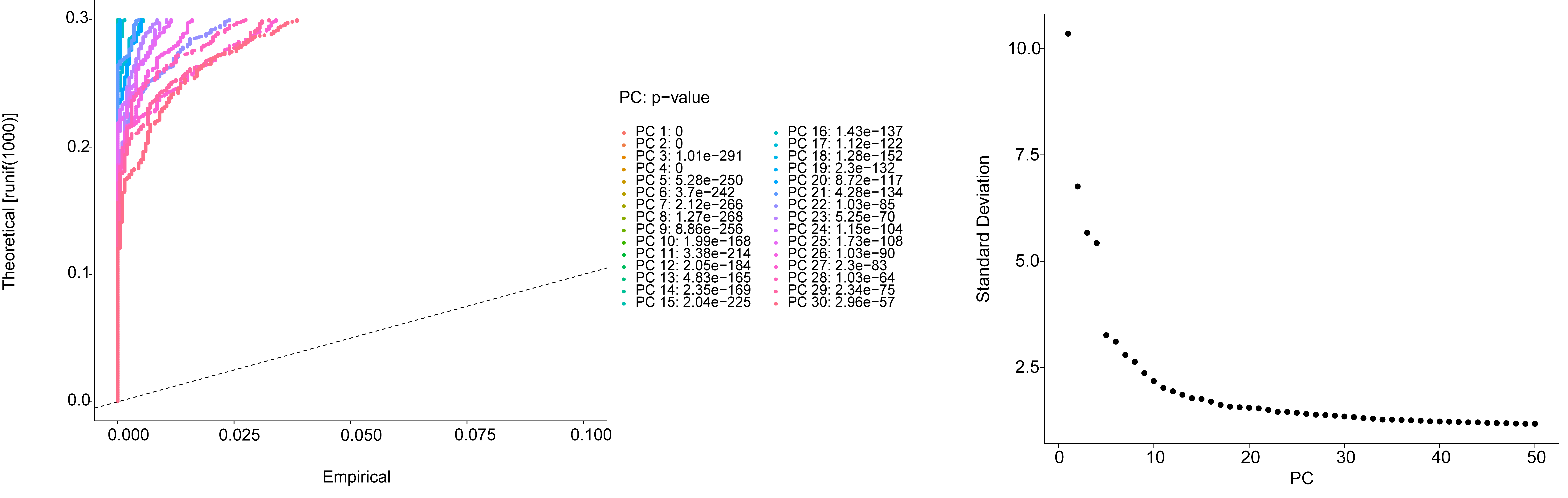

Supplement: S6 Fig — (TIF) [file pone.0343798.s006.tif]

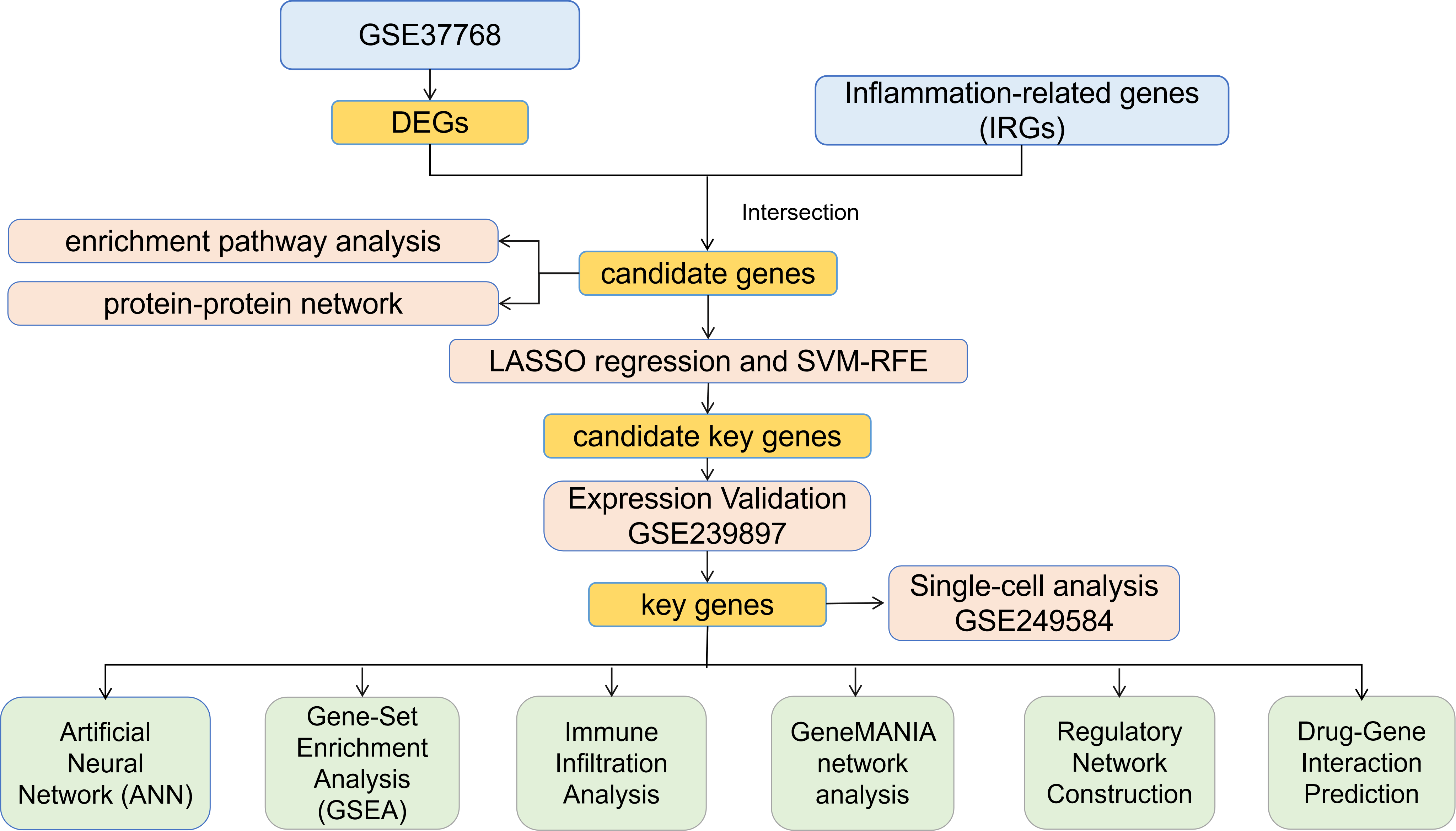

Supplement: S7 Fig — (TIF) [file pone.0343798.s007.tif]

Variable importance using connection weights

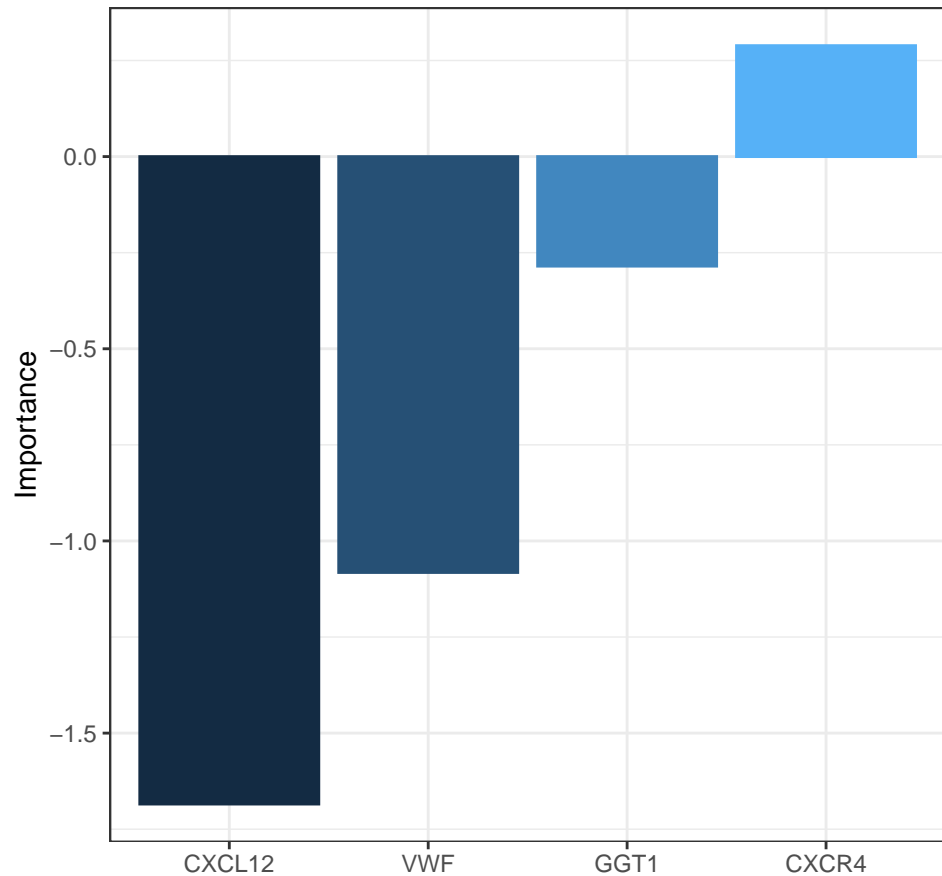

Supplement: S1 File — ssGSEA-Validation Set – Immune infiltration analysis of validation set. rstudio-export – Differences in cell number and proportion. (ZIP) [file pone.0343798.s012.zip › 04_ANN-/02.importance.pdf]

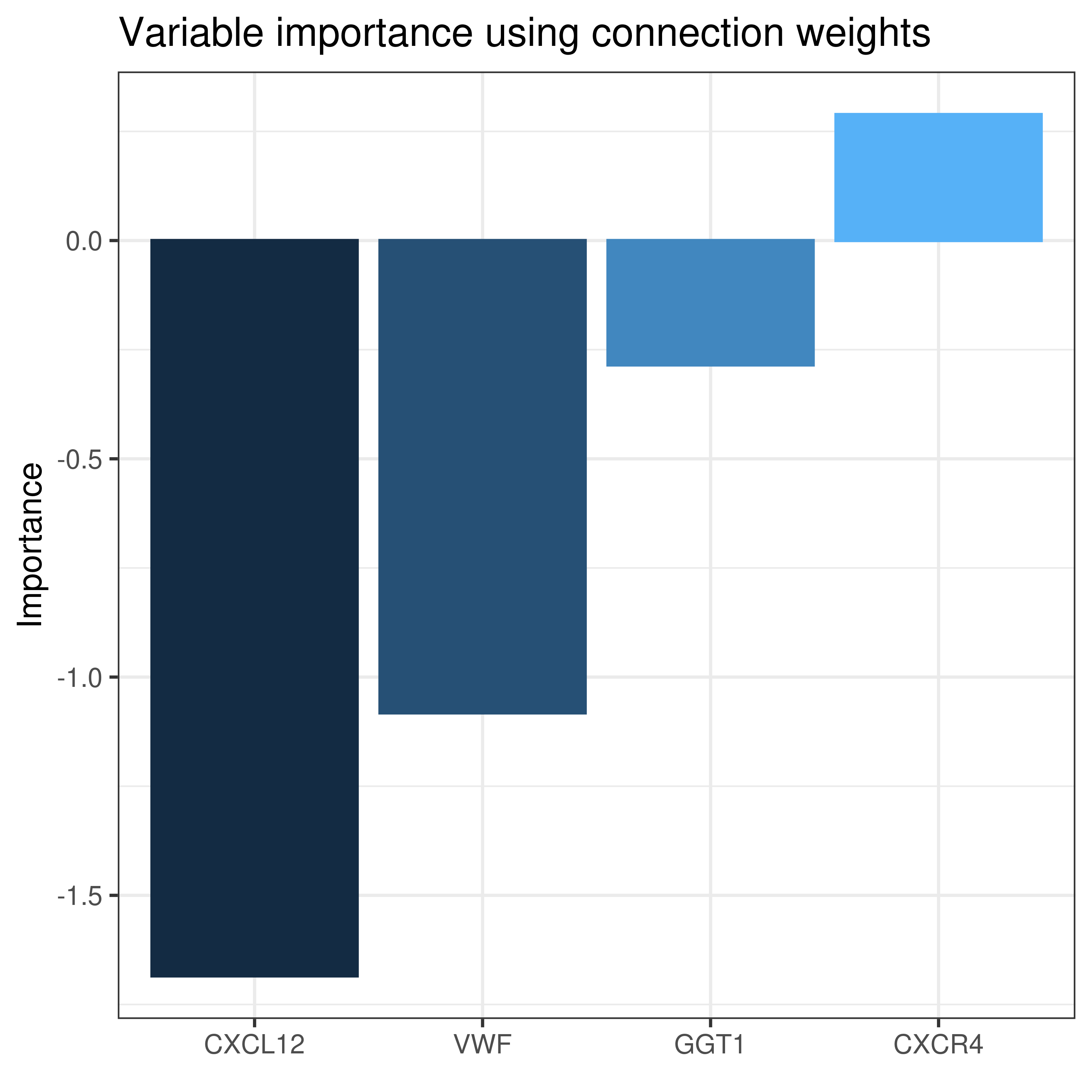

Supplement: S1 File — ssGSEA-Validation Set – Immune infiltration analysis of validation set. rstudio-export – Differences in cell number and proportion. (ZIP) [file pone.0343798.s012.zip › 04_ANN-/02.importance.png]

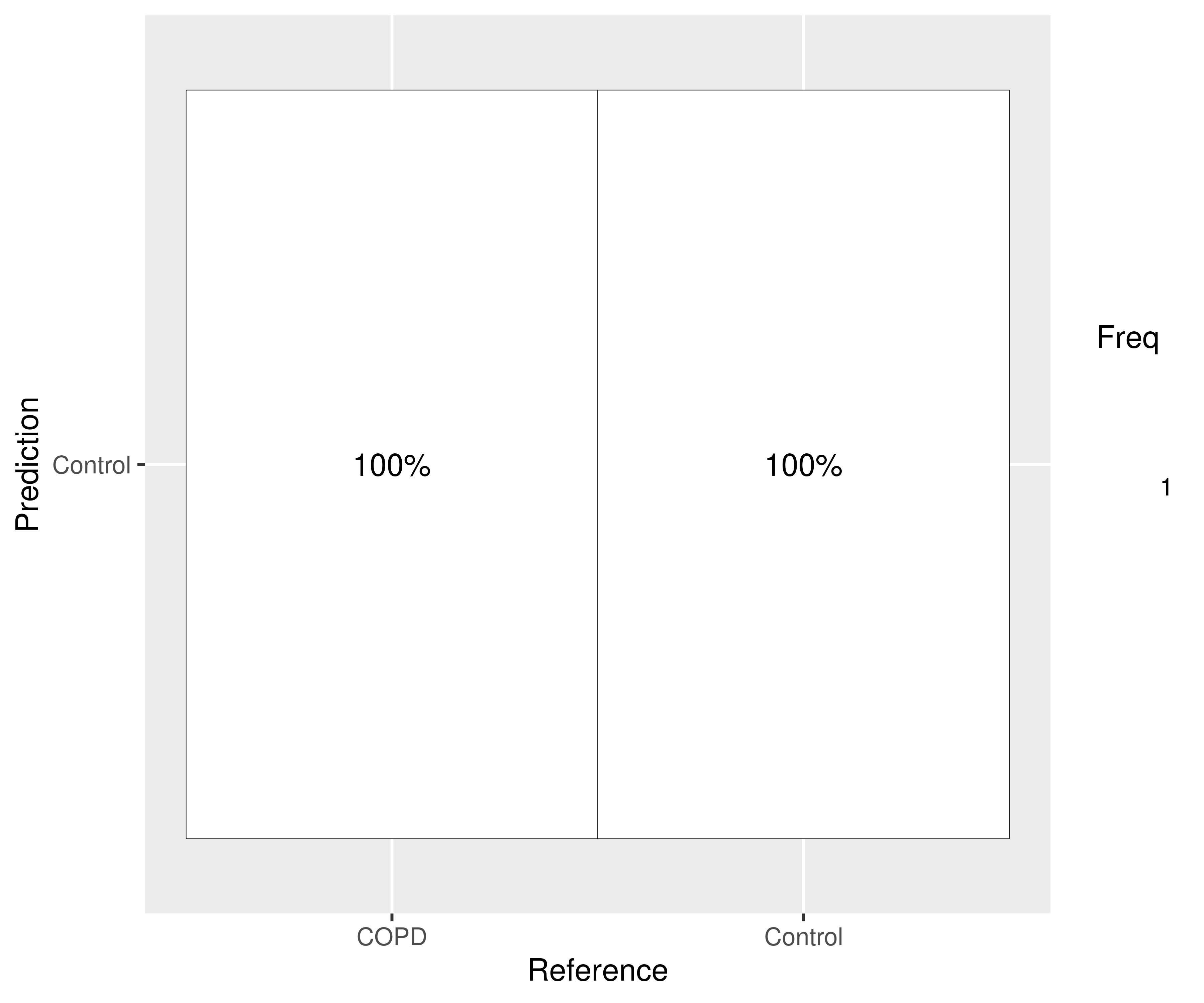

Supplement: S1 File — ssGSEA-Validation Set – Immune infiltration analysis of validation set. rstudio-export – Differences in cell number and proportion. (ZIP) [file pone.0343798.s012.zip › 04_ANN-/03.confusion_matrix.png]

# Training Set ROC Curve

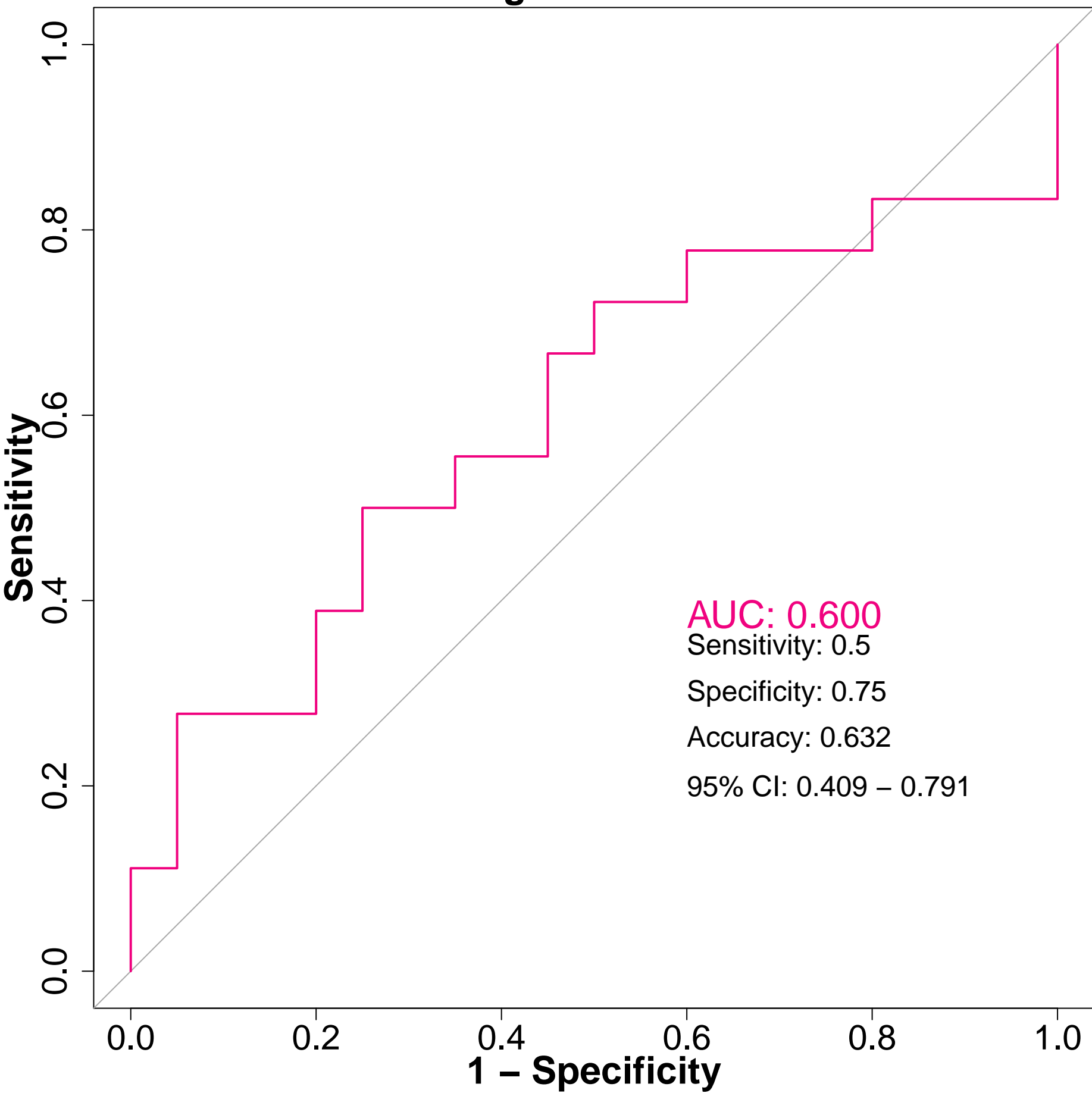

Supplement: S1 File — ssGSEA-Validation Set – Immune infiltration analysis of validation set. rstudio-export – Differences in cell number and proportion. (ZIP) [file pone.0343798.s012.zip › 04_ANN-/04.roc.pdf]

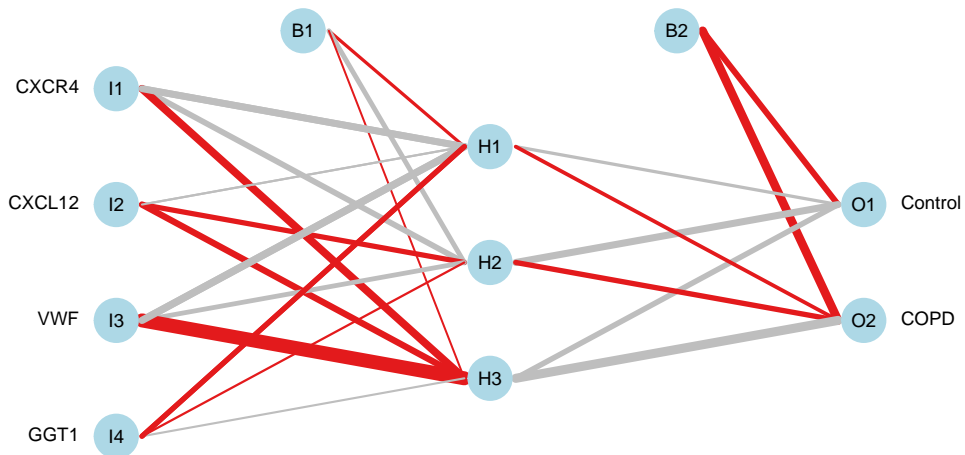

Supplement: S1 File — ssGSEA-Validation Set – Immune infiltration analysis of validation set. rstudio-export – Differences in cell number and proportion. (ZIP) [file pone.0343798.s012.zip › 04_ANN-/01.ANN.pdf]

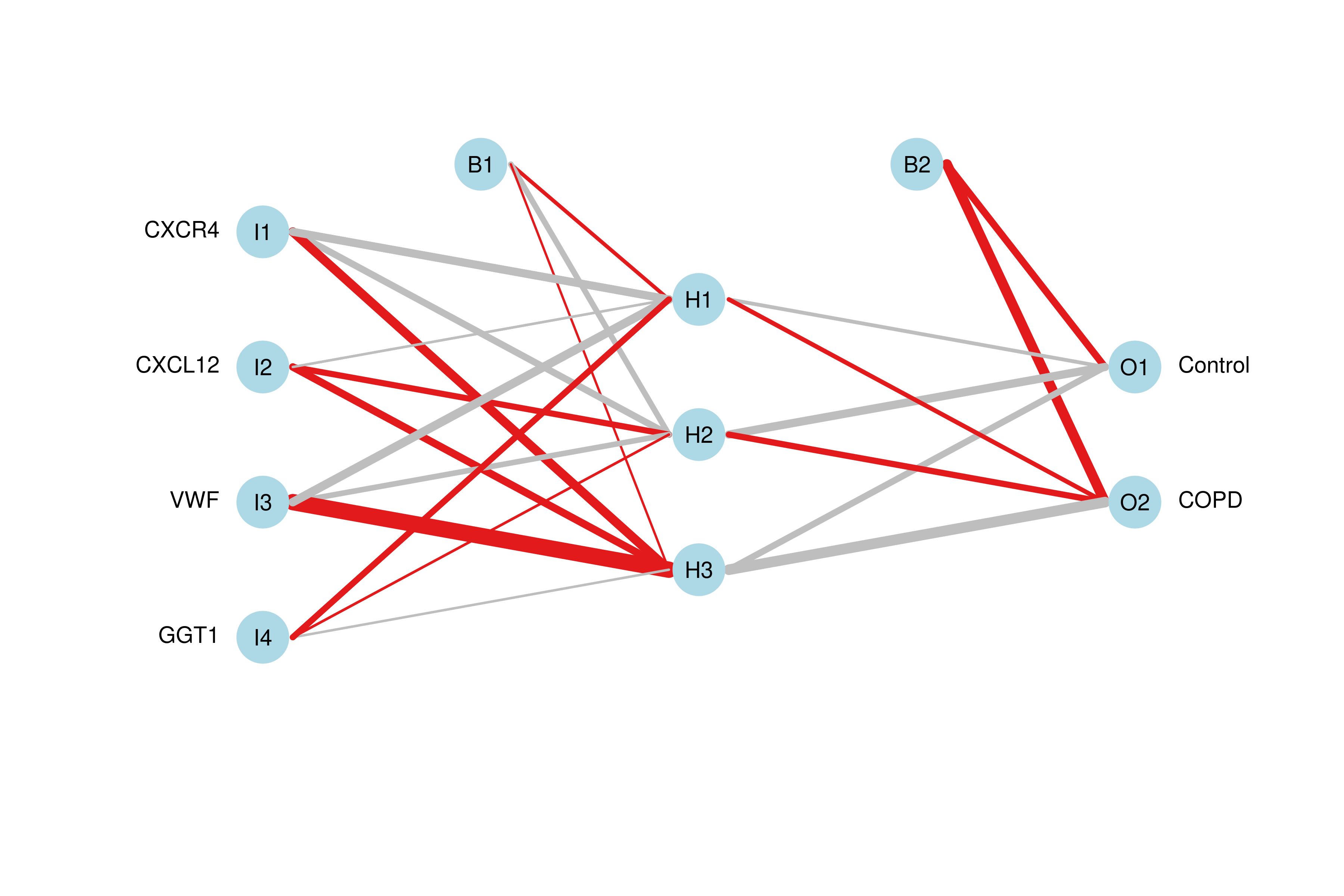

Supplement: S1 File — ssGSEA-Validation Set – Immune infiltration analysis of validation set. rstudio-export – Differences in cell number and proportion. (ZIP) [file pone.0343798.s012.zip › 04_ANN-/01.ANN.png]

# Validation Set ROC Curve

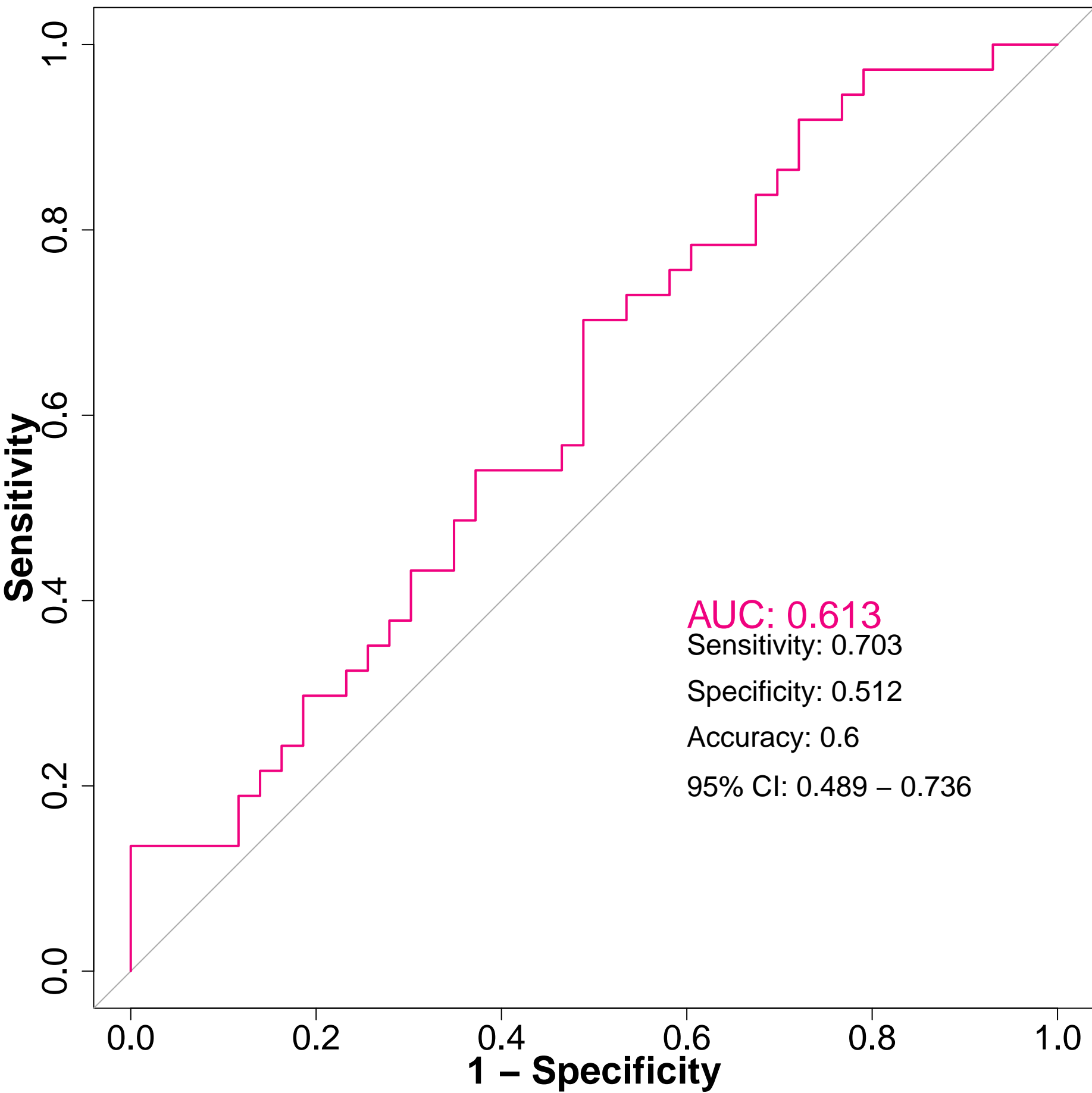

Supplement: S1 File — ssGSEA-Validation Set – Immune infiltration analysis of validation set. rstudio-export – Differences in cell number and proportion. (ZIP) [file pone.0343798.s012.zip › 04_ANN-/08.roc_ver.pdf]

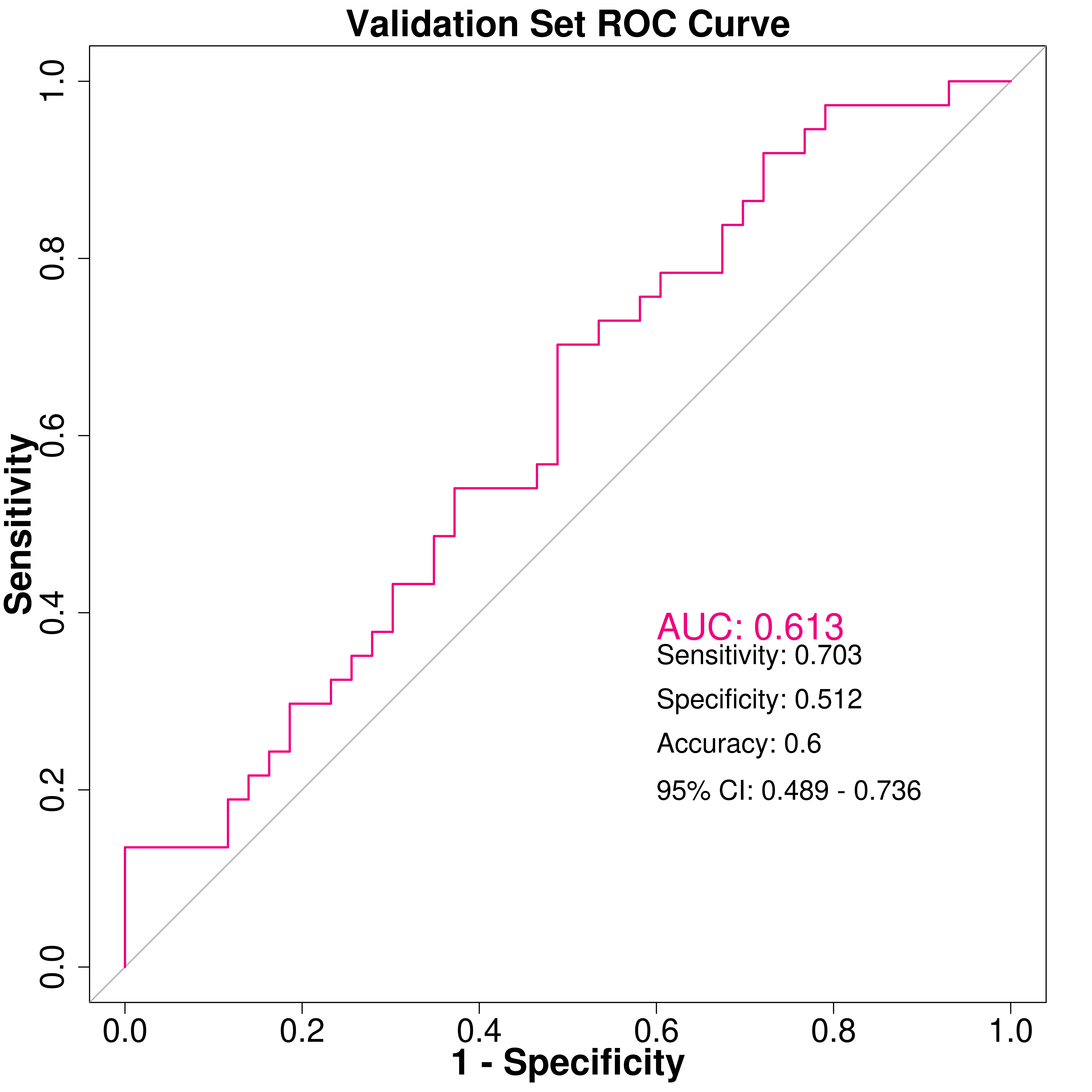

Supplement: S1 File — ssGSEA-Validation Set – Immune infiltration analysis of validation set. rstudio-export – Differences in cell number and proportion. (ZIP) [file pone.0343798.s012.zip › 04_ANN-/08.roc_ver.png]

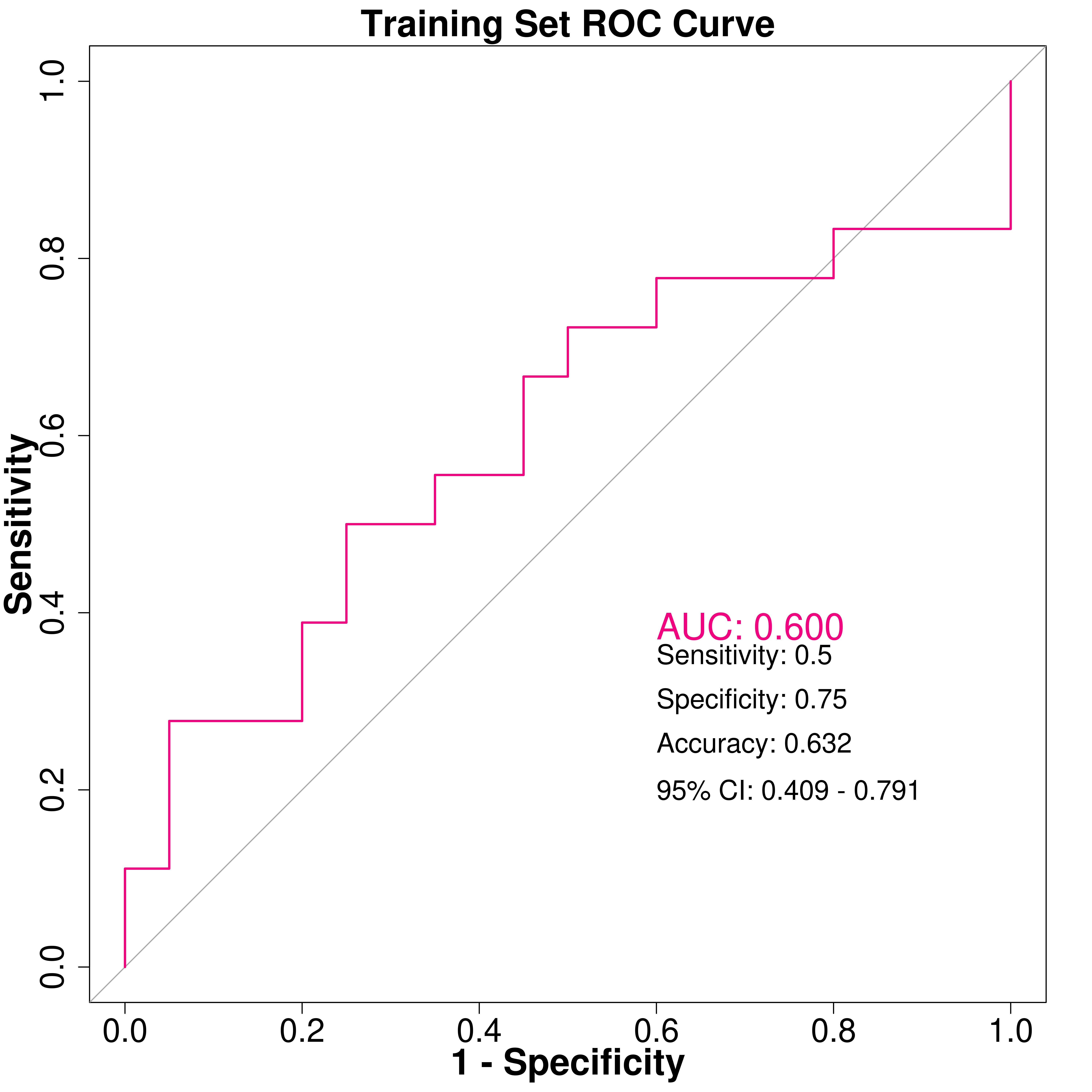

Supplement: S1 File — ssGSEA-Validation Set – Immune infiltration analysis of validation set. rstudio-export – Differences in cell number and proportion. (ZIP) [file pone.0343798.s012.zip › 04_ANN-/04.roc.png]

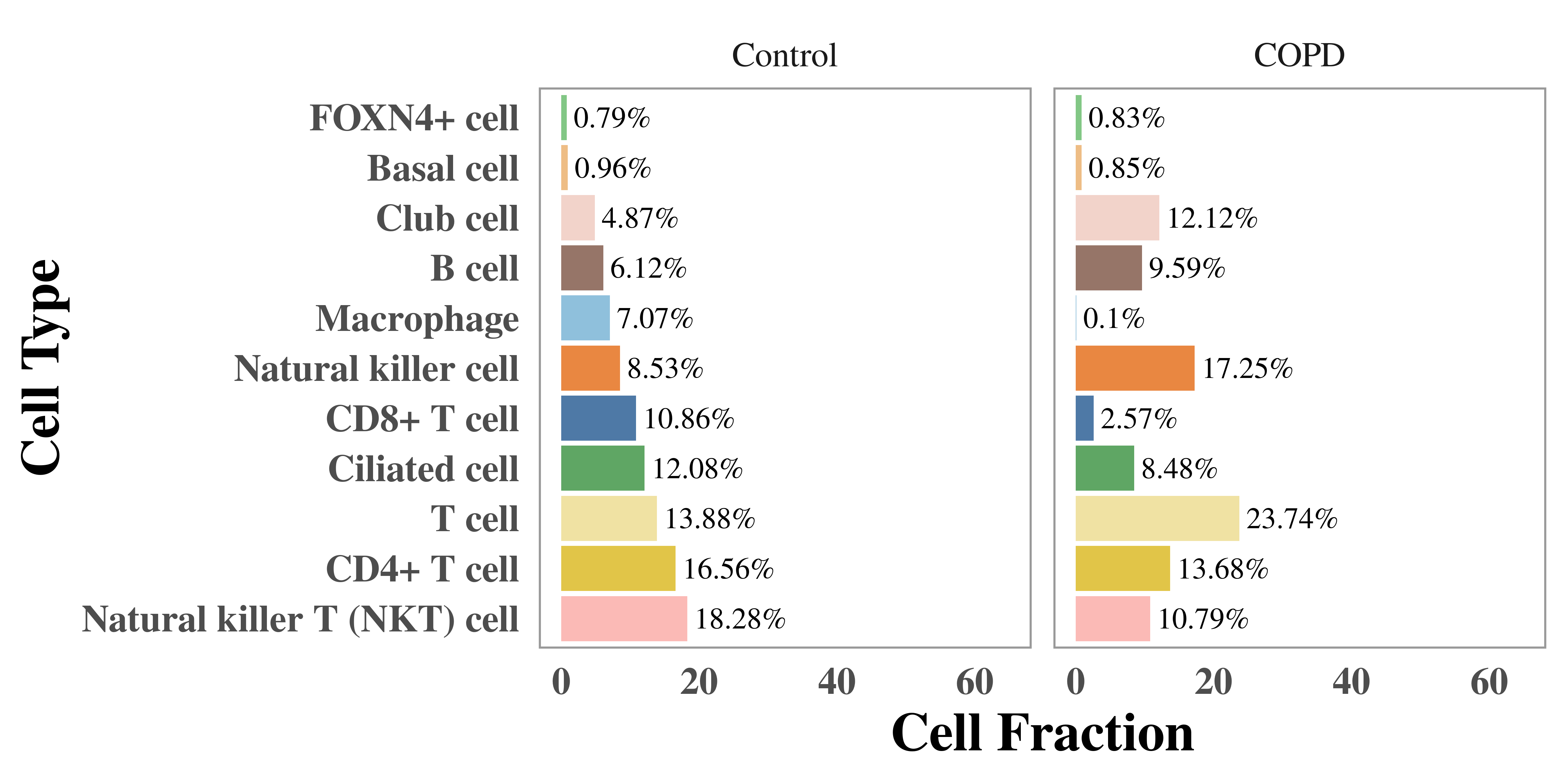

Supplement: S2 File — (ZIP) [file pone.0343798.s013.zip › 07.CellProportion.png]

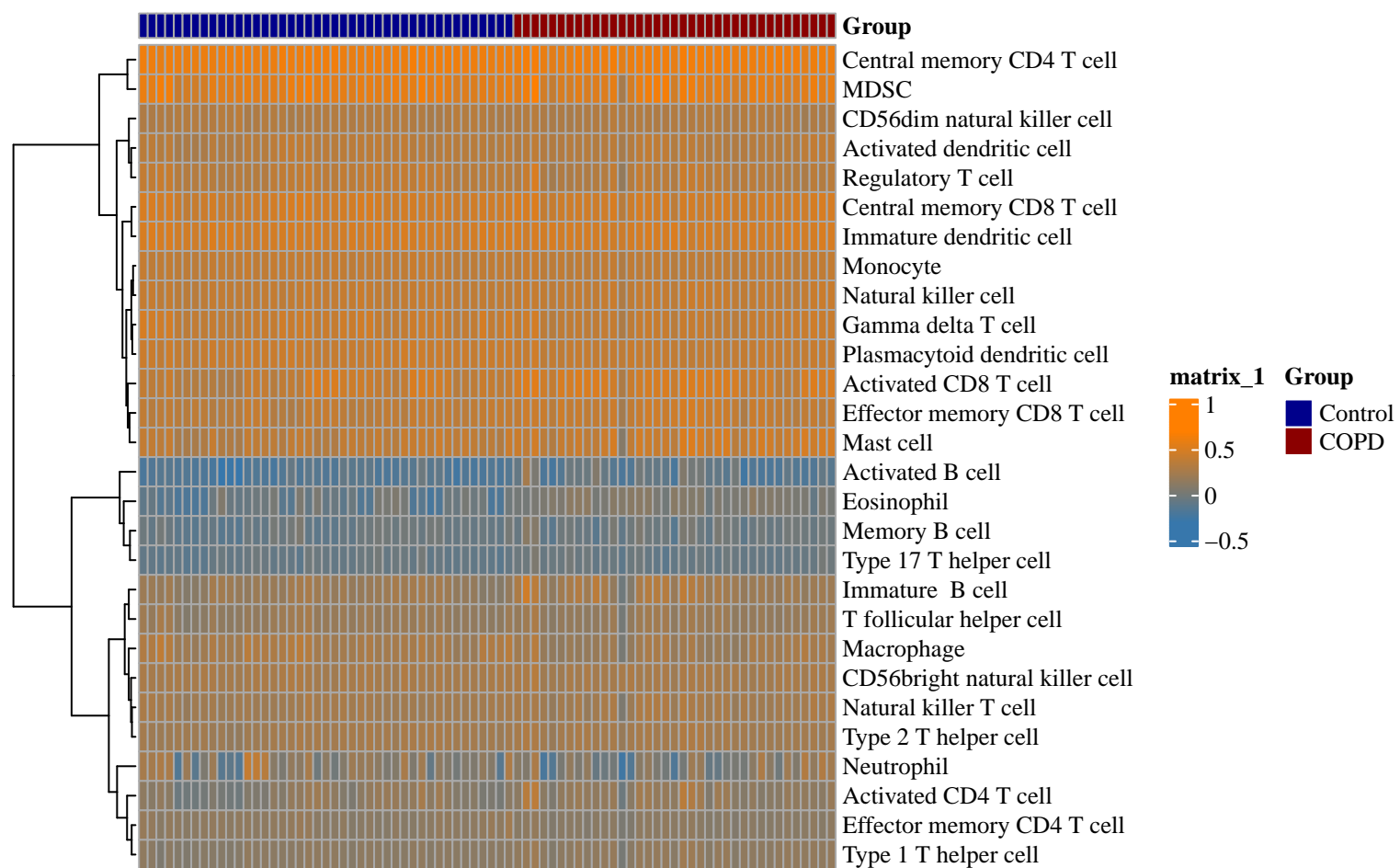

Supplement: S3 File — (ZIP) [file pone.0343798.s014.zip › ssGSEA-Validation Set/ssGSEA-Validation Set/01.heatmap.pdf]

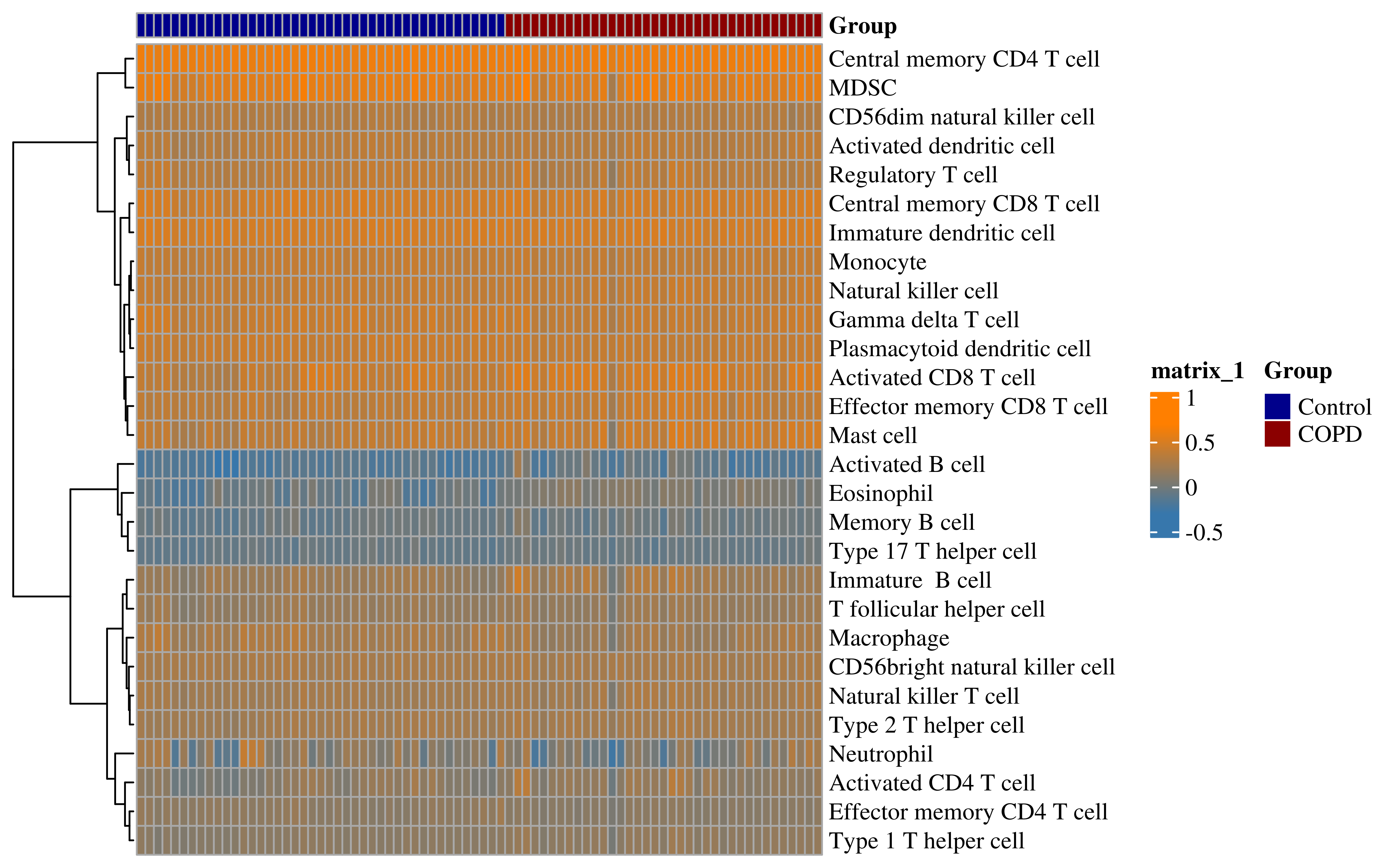

Supplement: S3 File — (ZIP) [file pone.0343798.s014.zip › ssGSEA-Validation Set/ssGSEA-Validation Set/01.heatmap.png]

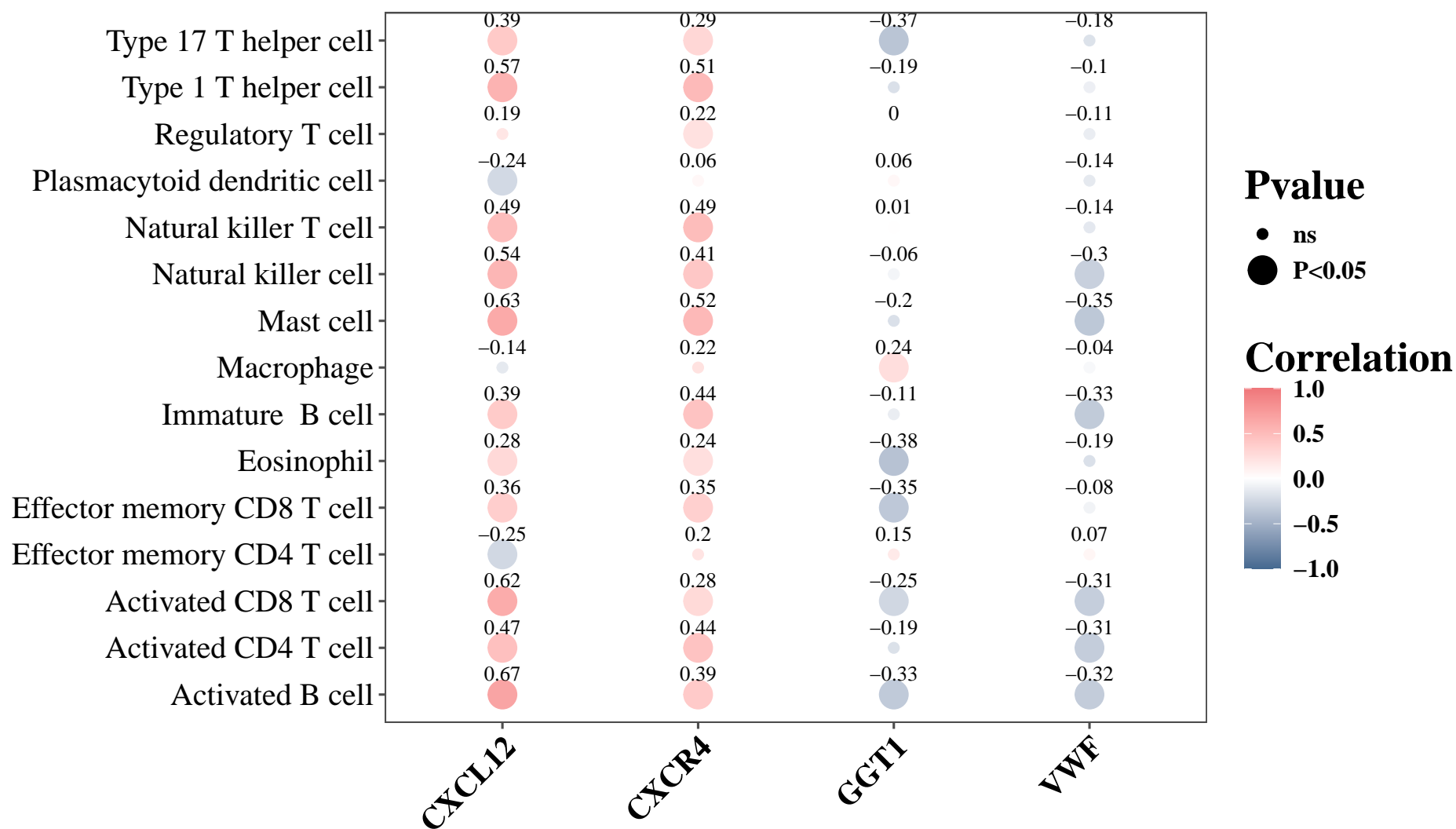

Supplement: S3 File — (ZIP) [file pone.0343798.s014.zip › ssGSEA-Validation Set/ssGSEA-Validation Set/02.ssgsea_Box.pdf]

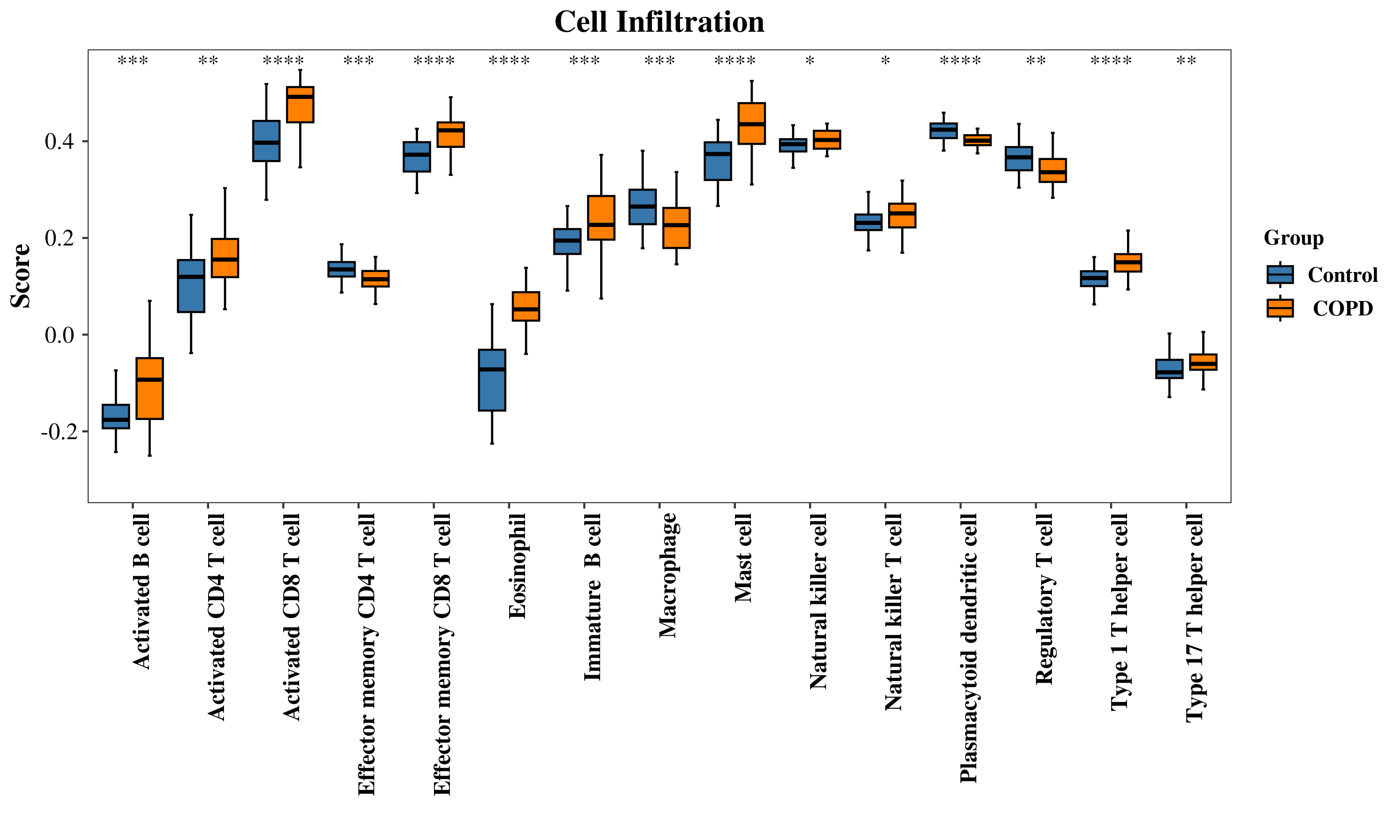

Supplement: S3 File — (ZIP) [file pone.0343798.s014.zip › ssGSEA-Validation Set/ssGSEA-Validation Set/02.ssgsea_Box.png]

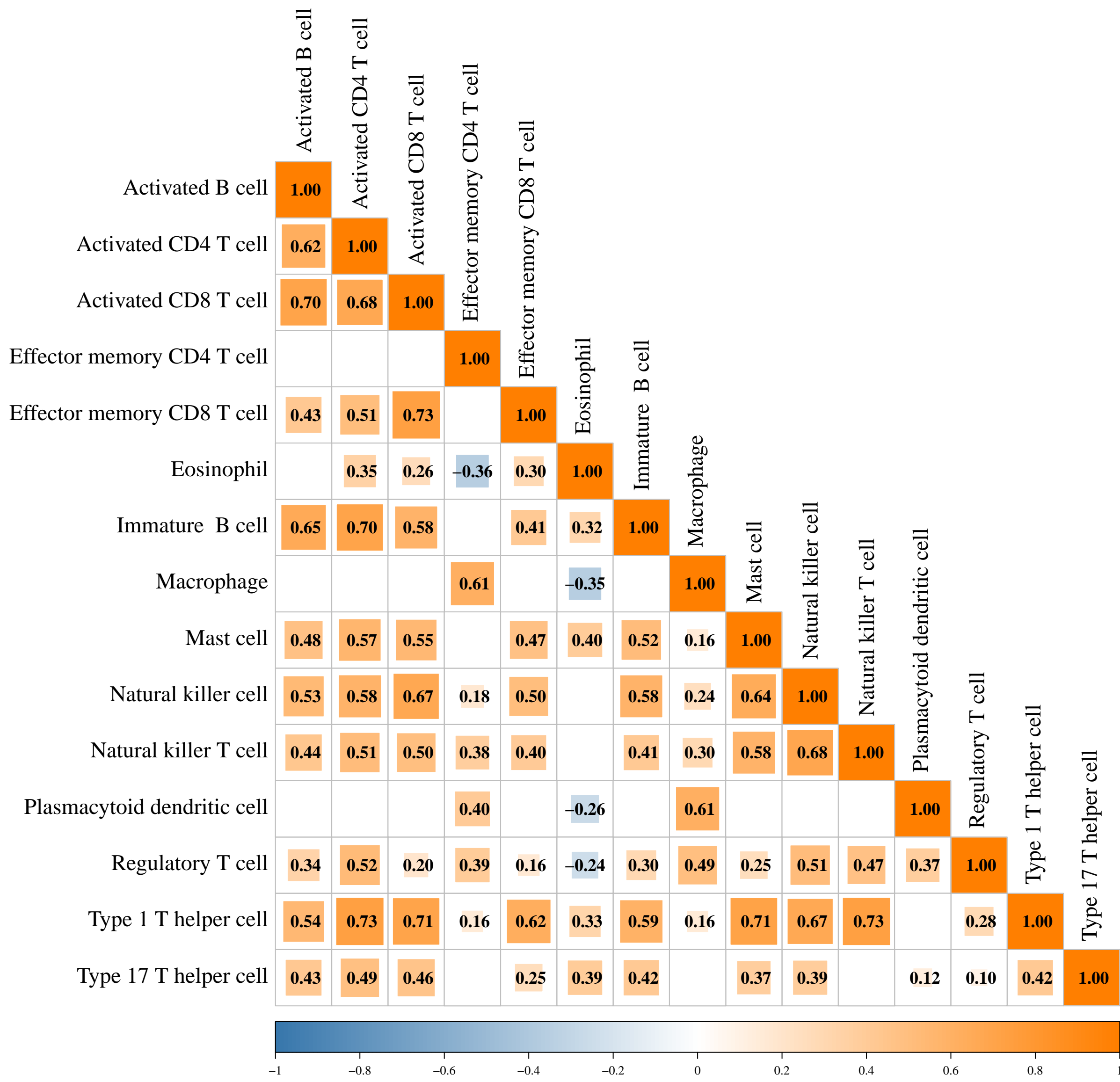

Supplement: S3 File — (ZIP) [file pone.0343798.s014.zip › ssGSEA-Validation Set/ssGSEA-Validation Set/03.cell_corHeatmap.pdf]

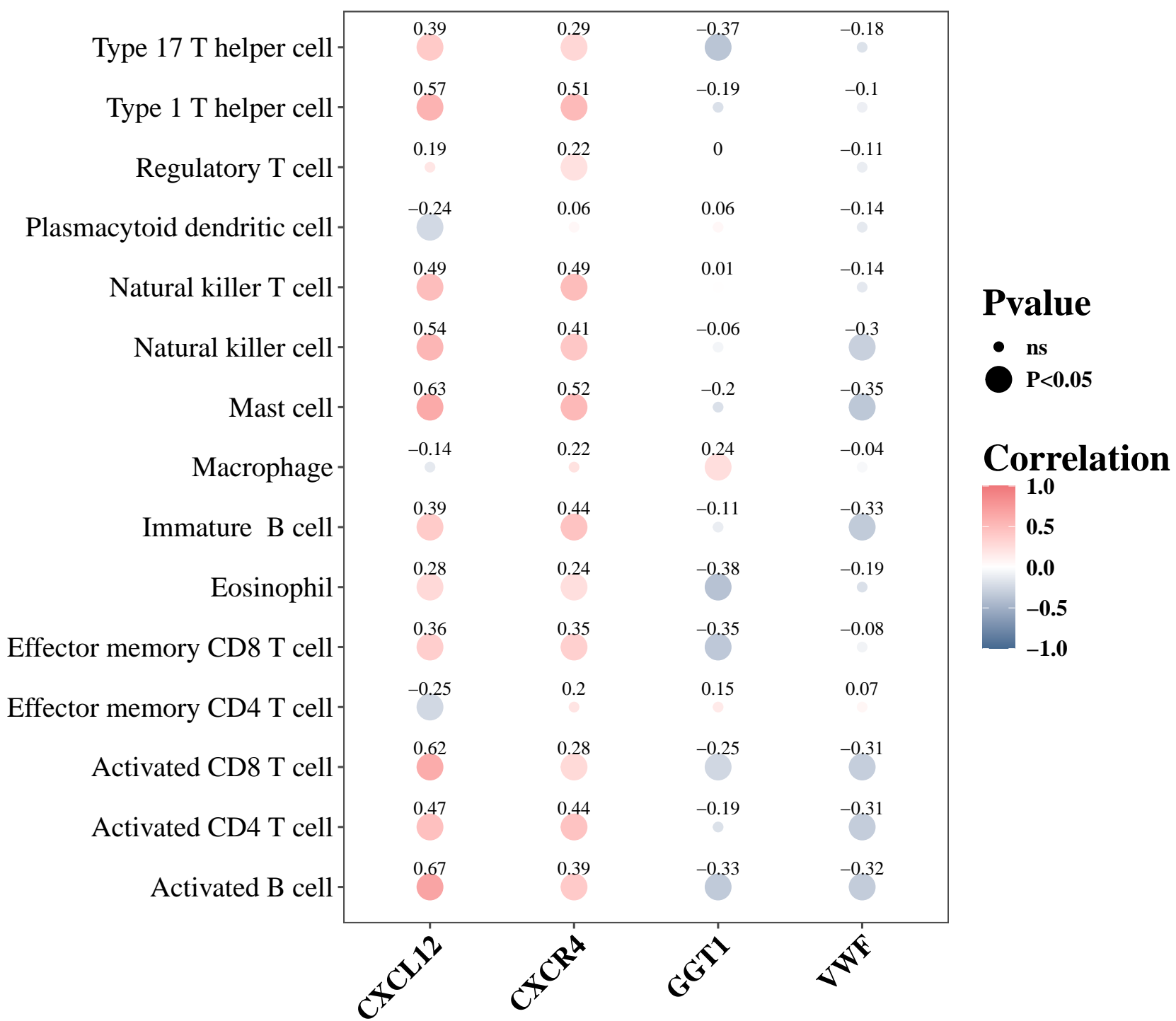

Supplement: S3 File — (ZIP) [file pone.0343798.s014.zip › ssGSEA-Validation Set/ssGSEA-Validation Set/04.correlation.pdf]

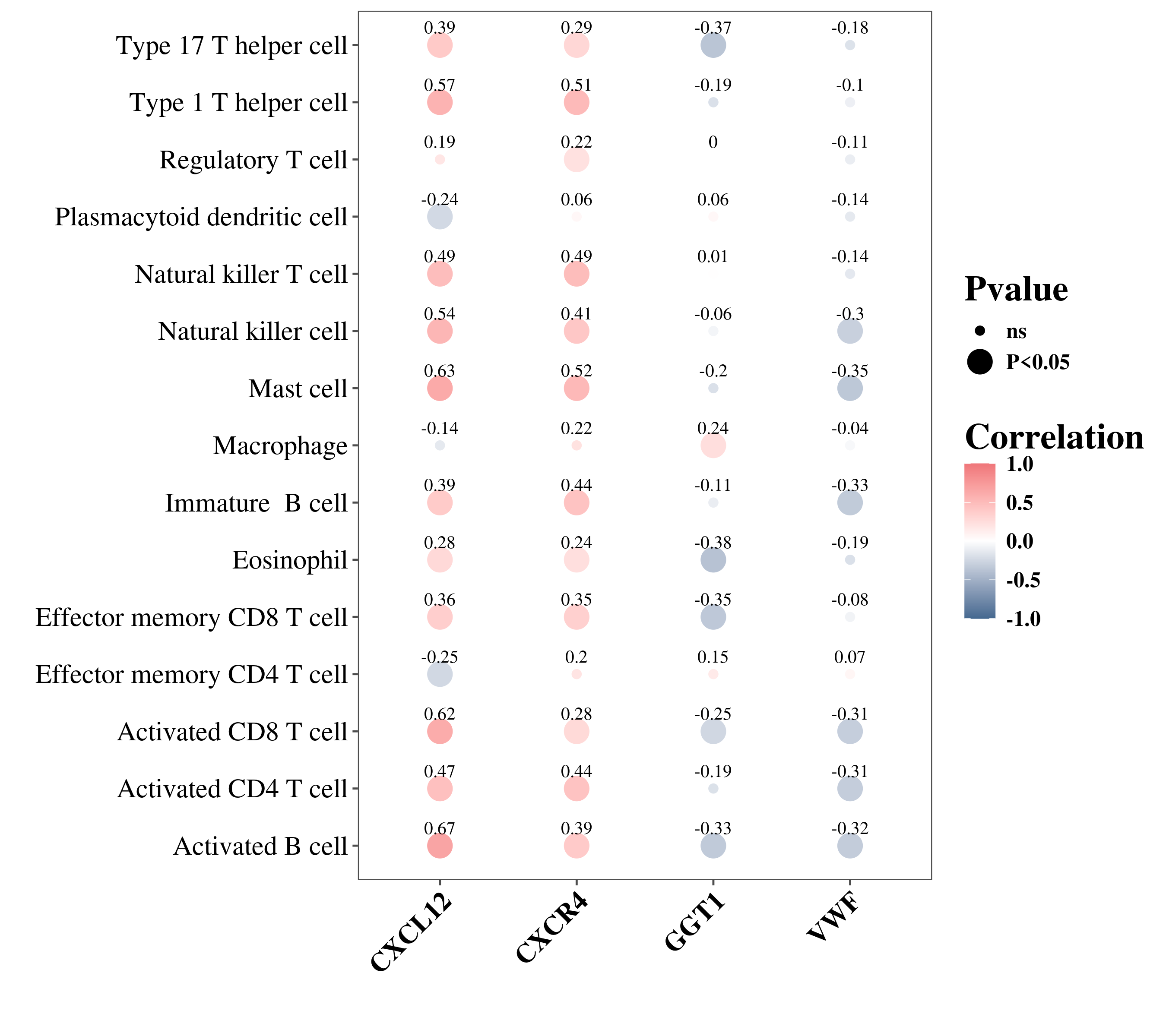

Supplement: S3 File — (ZIP) [file pone.0343798.s014.zip › ssGSEA-Validation Set/ssGSEA-Validation Set/04.correlation.png]
