# Supplementary material for: Based on single-cell and transcriptome analysis of inflammatory pathway biomarkers and their molecular mechanisms in chronic obstructive pulmonary disease
Source: PLoS One. 2026 Feb 25;21(2):e0343798. doi: 10.1371/journal.pone.0343798 (PMC12935203; doi:10.1371/journal.pone.0343798)
Supplement: S1 File — ssGSEA-Validation Set – Immune infiltration analysis of validation set. rstudio-export – Differences in cell number and proportion. (ZIP) [file pone.0343798.s012.zip › 04_ANN-/07.confusion_matrix_ver.pdf]

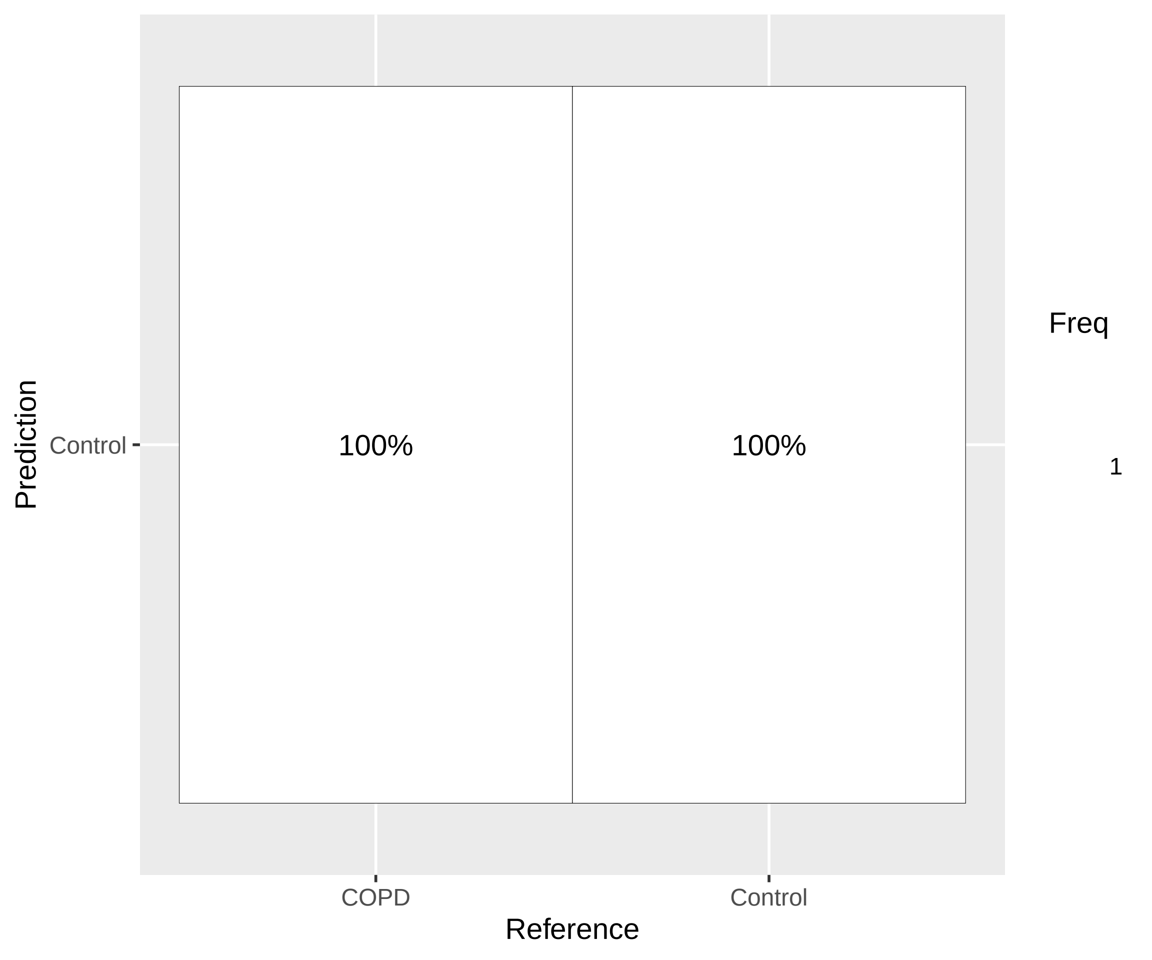

Prediction
Control
100%
100%
Freq
1
COPD
Control
Reference
