# Supplementary material for: Based on single-cell and transcriptome analysis of inflammatory pathway biomarkers and their molecular mechanisms in chronic obstructive pulmonary disease
Source: PLoS One. 2026 Feb 25;21(2):e0343798. doi: 10.1371/journal.pone.0343798 (PMC12935203; doi:10.1371/journal.pone.0343798)
Supplement: S2 File — (ZIP) [file pone.0343798.s013.zip › 07.CellProportion.pdf]

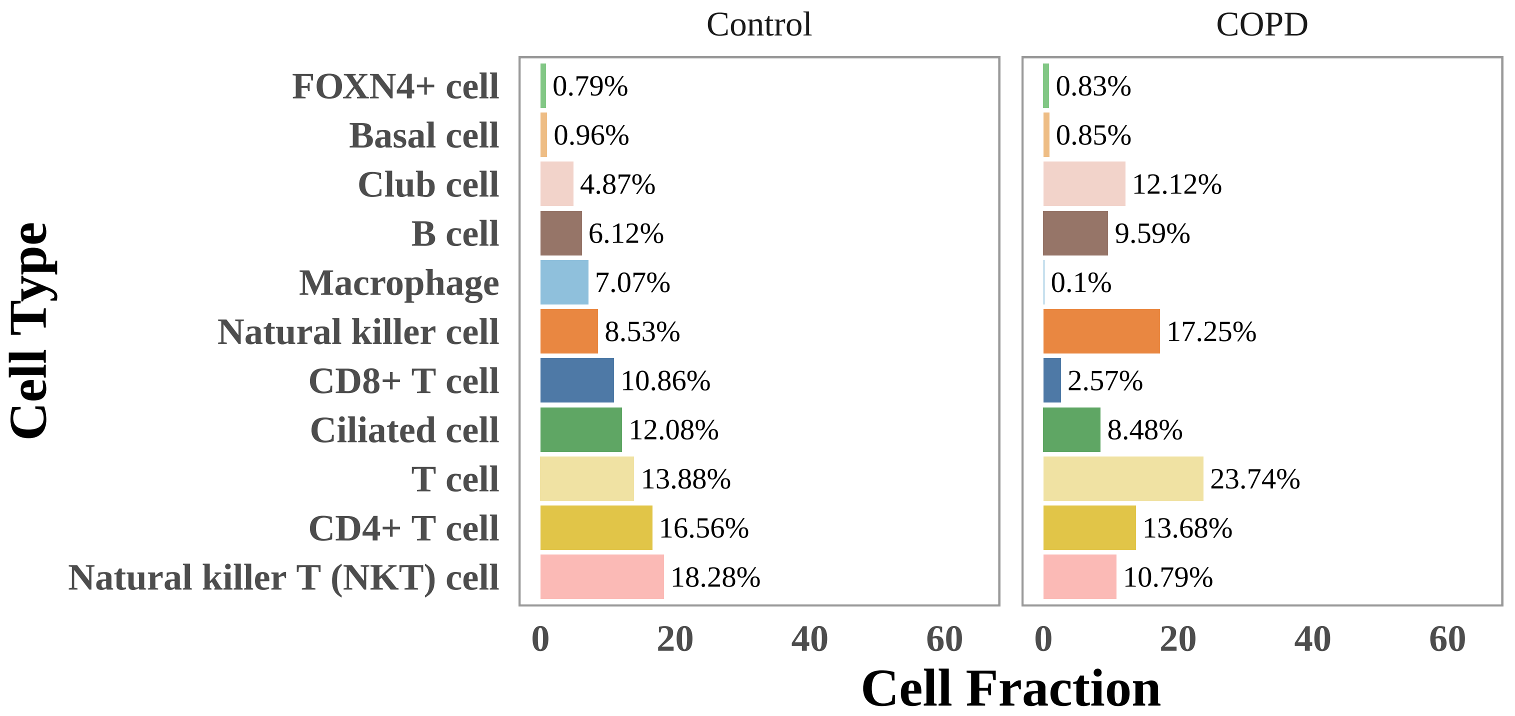
Control
COPD
Cell Type
FOXN4+ cell
Basal cell
Club cell
B cell
Macrophage
Natural killer cell
CD8+ T cell
Ciliated cell
T cell
CD4+ T cell
Natural killer T (NKT) cell
0.79%
0.96%
4.87%
6.12%
7.07%
8.53%
10.86%
12.08%
13.88%
16.56%
18.28%
0.83%
0.85%
12.12%
9.59%
0.1%
17.25%
2.57%
8.48%
23.74%
13.68%
10.79%
0
20
40
60
0
20
40
60
Cell Fraction

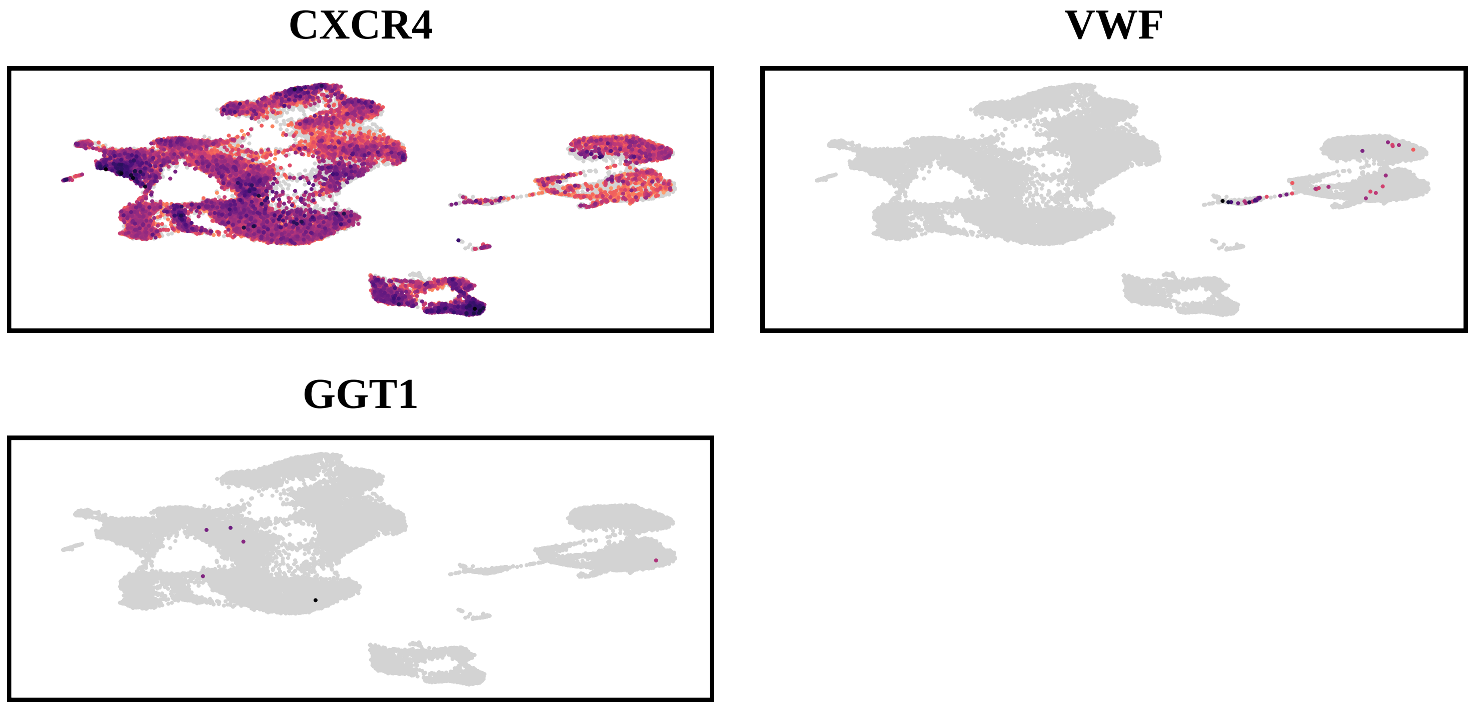
CXCR4
VWF
GGT1

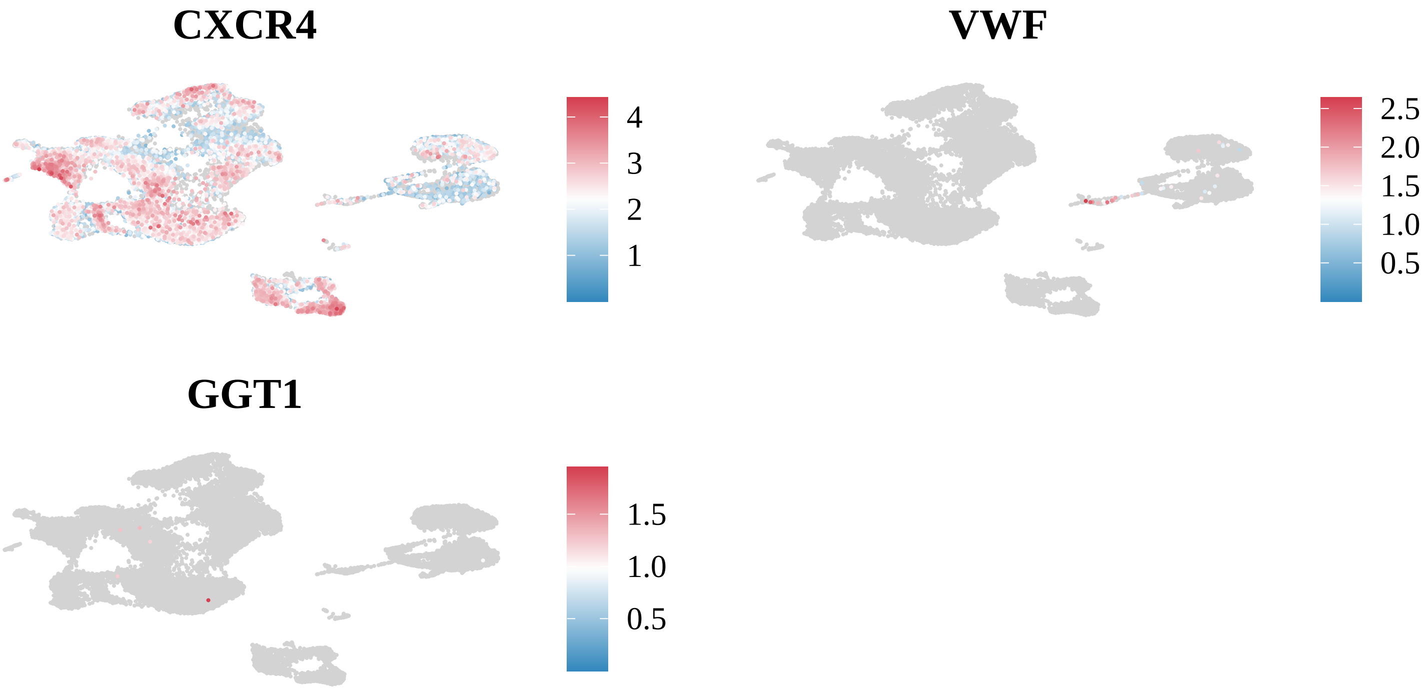
CXCR4
4
3
2
1
VWF
2.5
2.0
1.5
1.0
0.5
GGT1
1.5
1.0
0.5

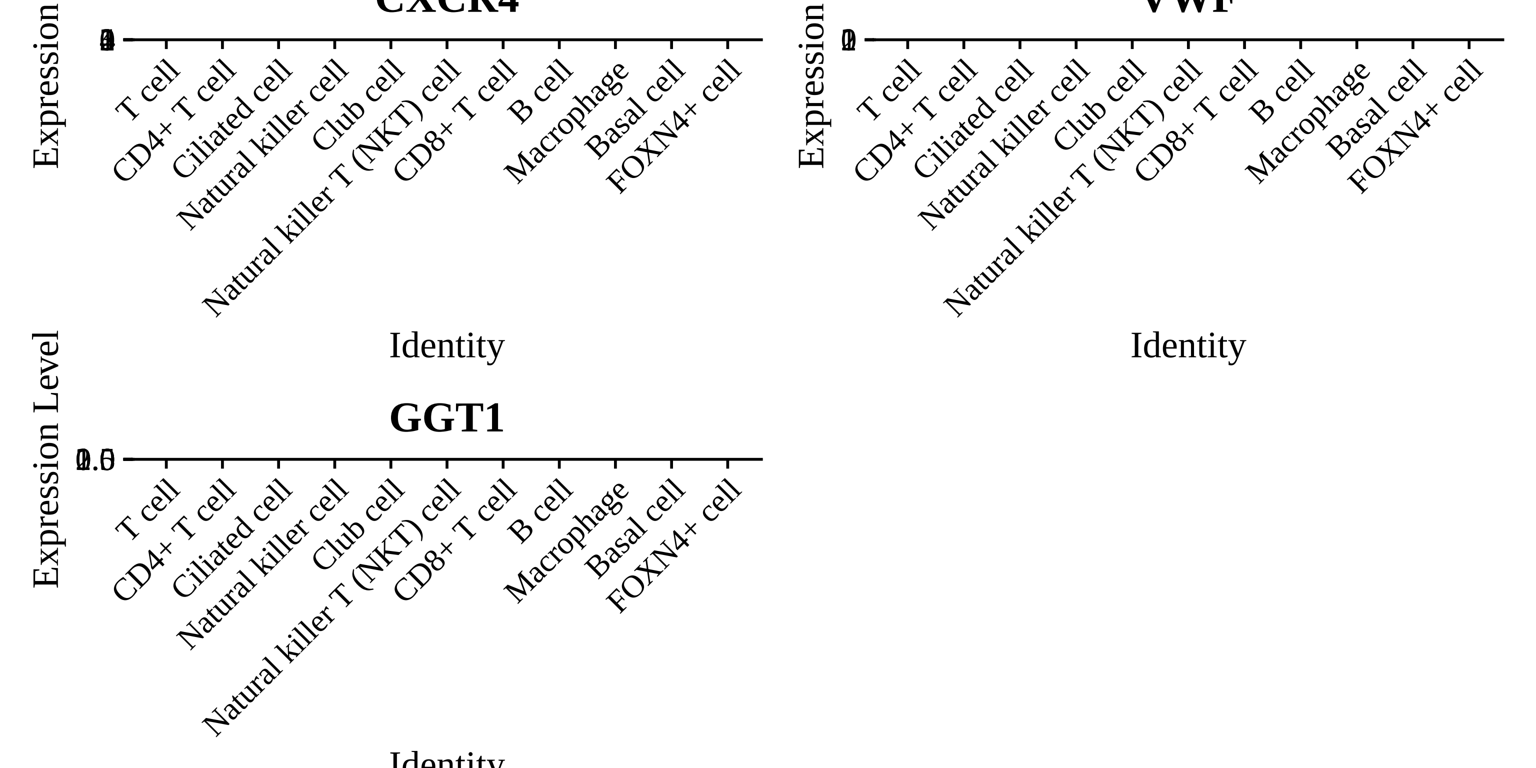
CXCR4
VWF
GGT1
Expression Level
T cell
CD4+ T cell
Ciliated cell
Natural killer cell
Club cell
Natural killer T (NKT) cell
CD8+ T cell
B cell
Macrophage
Basal cell
FOXN4+ cell
Identity

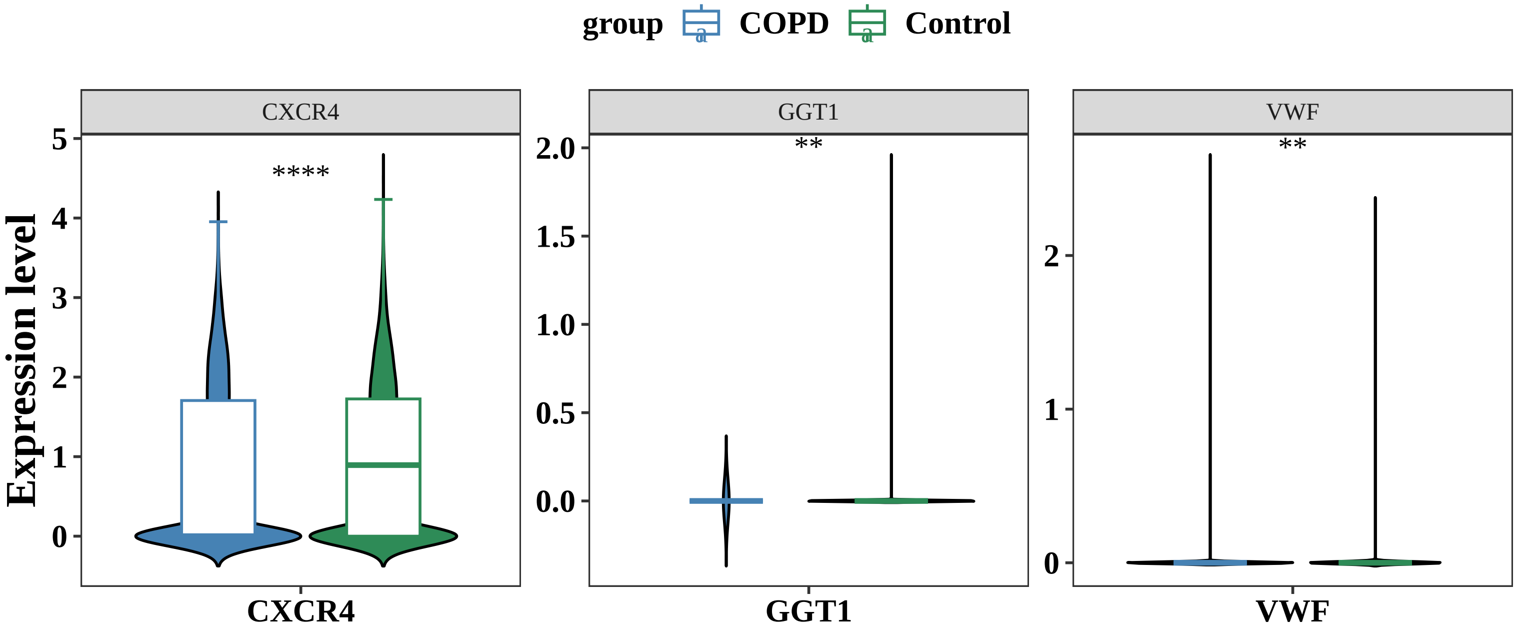
group
COPD
Control
CXCR4
GGT1
VWF
Expression level
****
**
**
0
1
2
3
4
5
0.0
0.5
1.0
1.5
2.0
0
1
2
CXCR4
GGT1
VWF

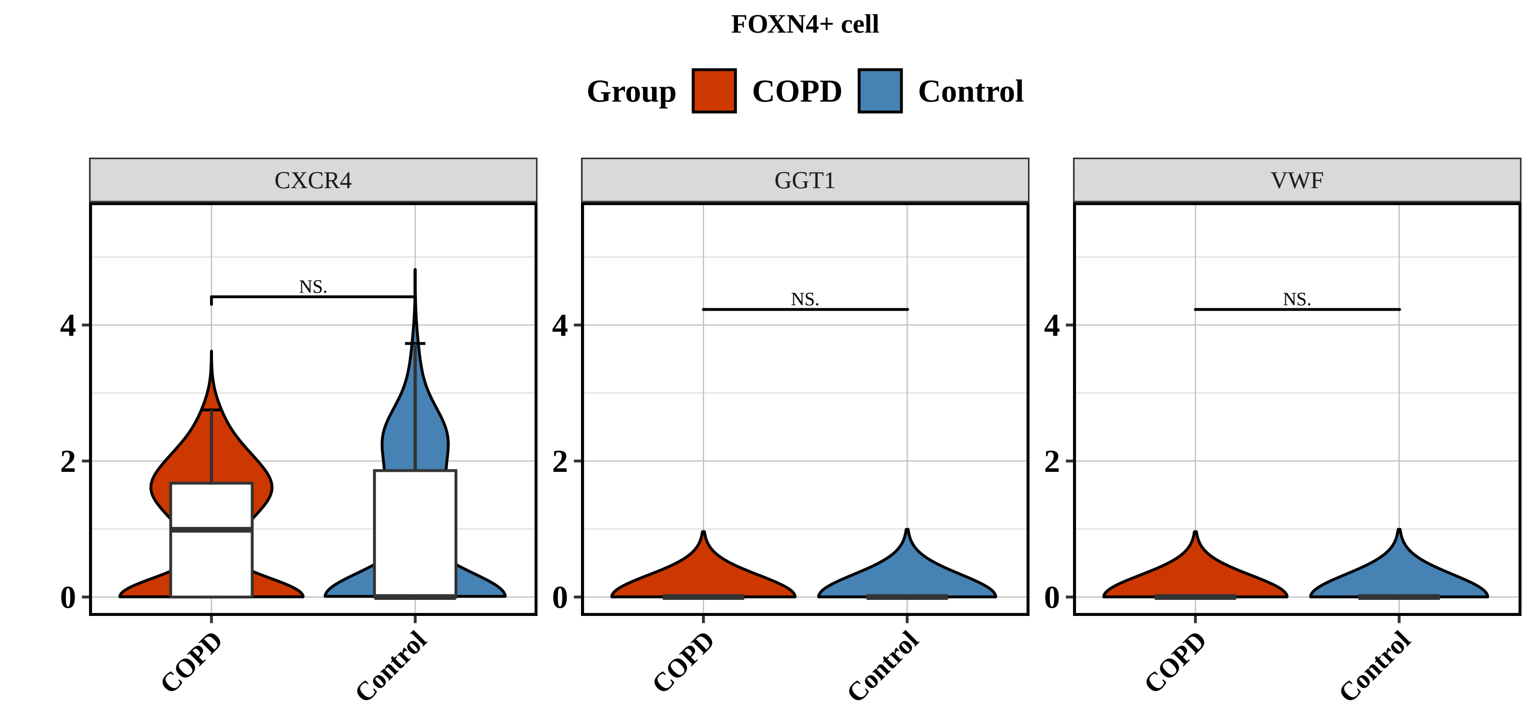
FOXN4+ cell
Group
COPD
Control
CXCR4
GGT1
VWF
NS.
NS.
NS.
0
2
4
COPD
Control

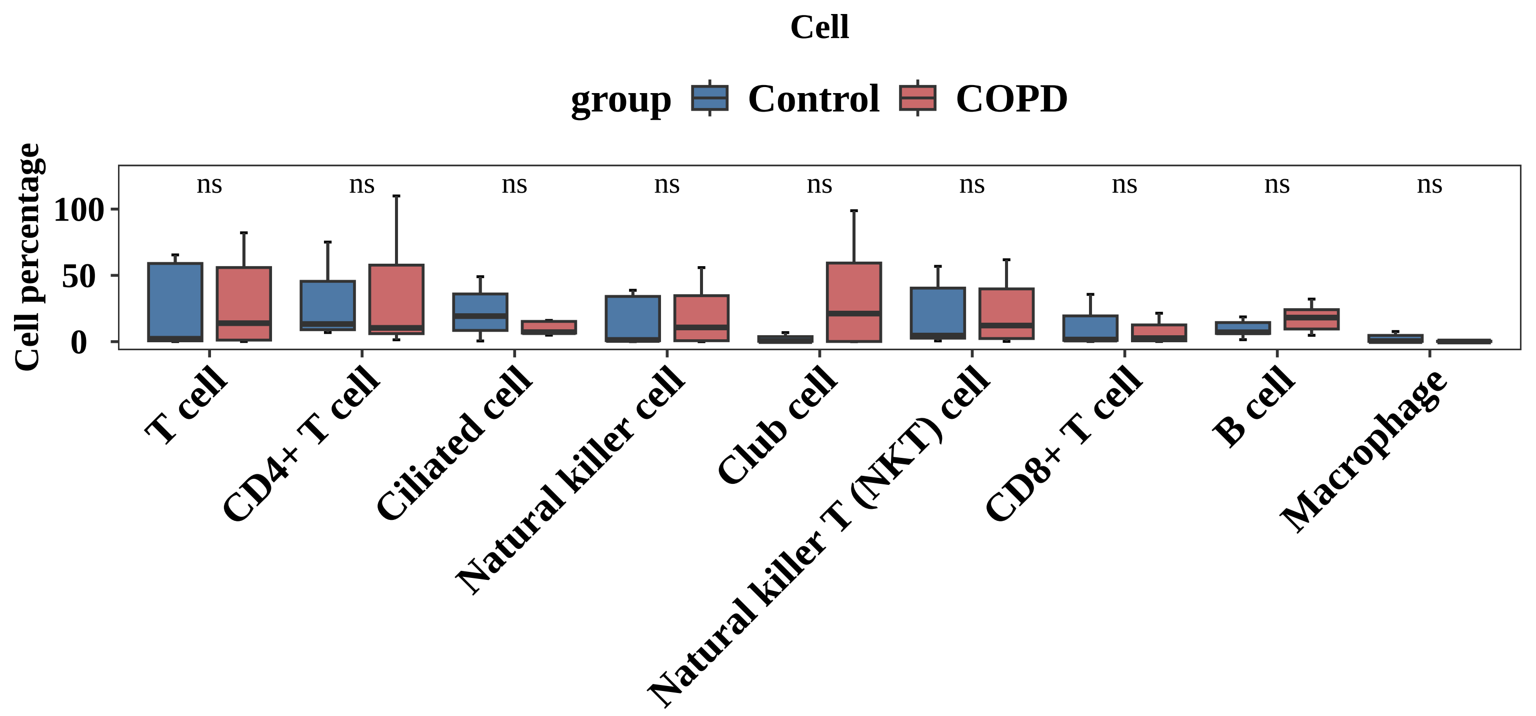

Cell
group
Control
COPD
Cell percentage
100
50
0
ns
T cell
CD4+ T cell
Ciliated cell
Natural killer cell
Club cell
Natural killer T (NKT) cell
CD8+ T cell
B cell
Macrophage

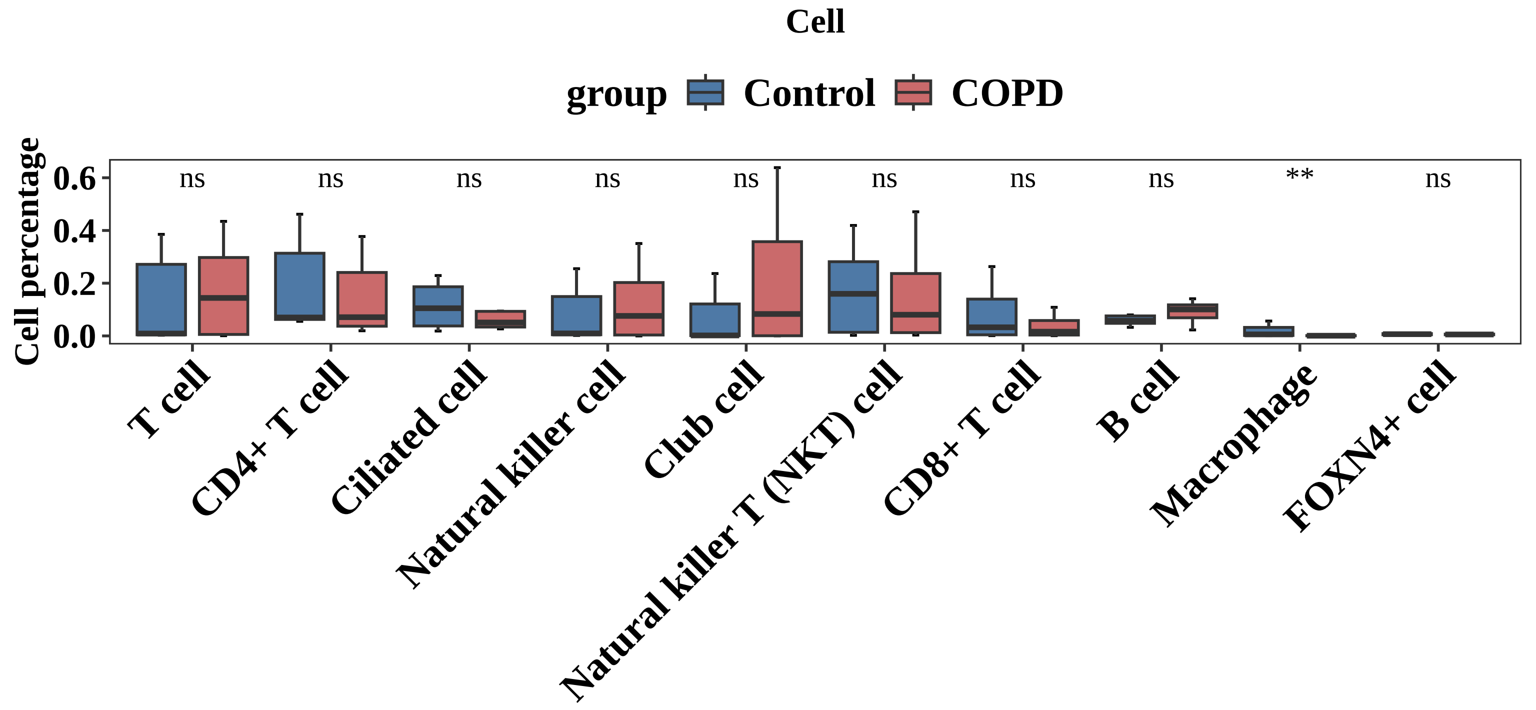
Cell
group
Control
COPD
Cell percentage
0.0
0.2
0.4
0.6
ns
ns
ns
ns
ns
ns
ns
ns
**
ns
T cell
CD4+ T cell
Ciliated cell
Natural killer cell
Club cell
Natural killer T (NKT) cell
CD8+ T cell
B cell
Macrophage
FOXN4+ cell

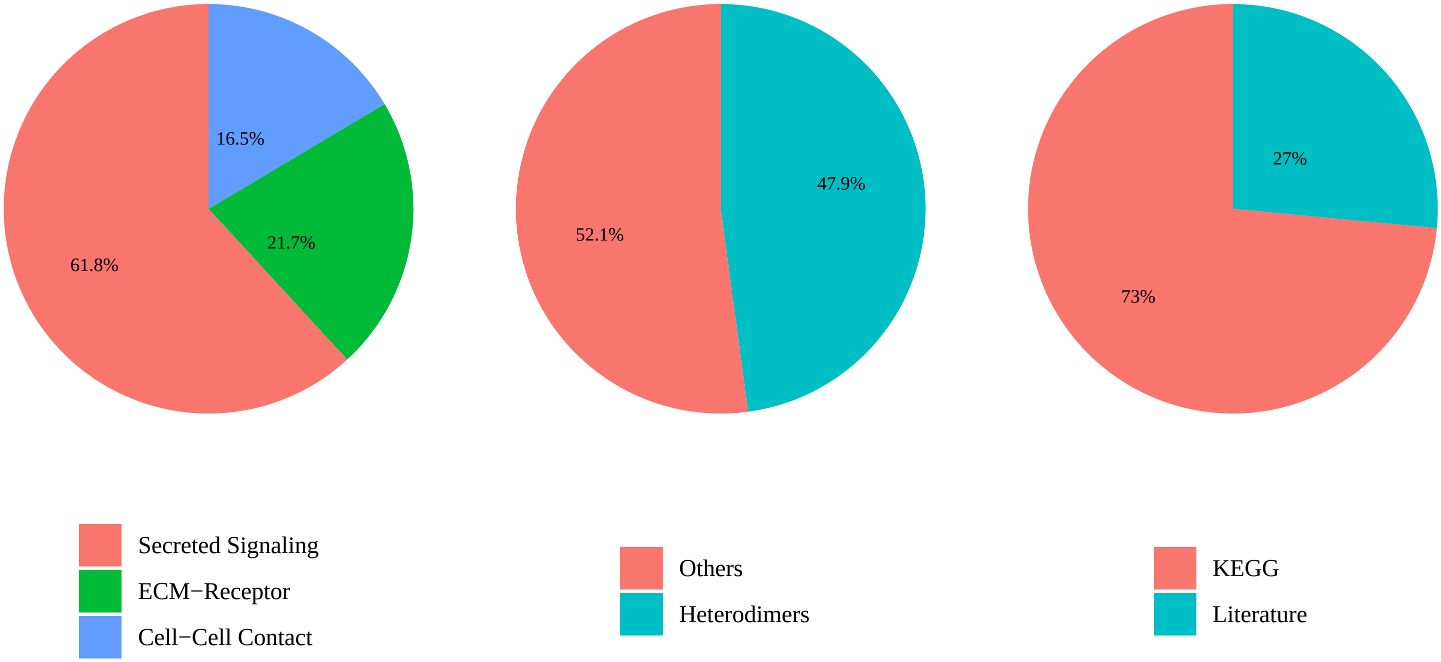

16.5%
21.7%
61.8%
47.9%
52.1%
27%
73%
Secreted Signaling
ECM−Receptor
Cell−Cell Contact
Others
Heterodimers
KEGG
Literature

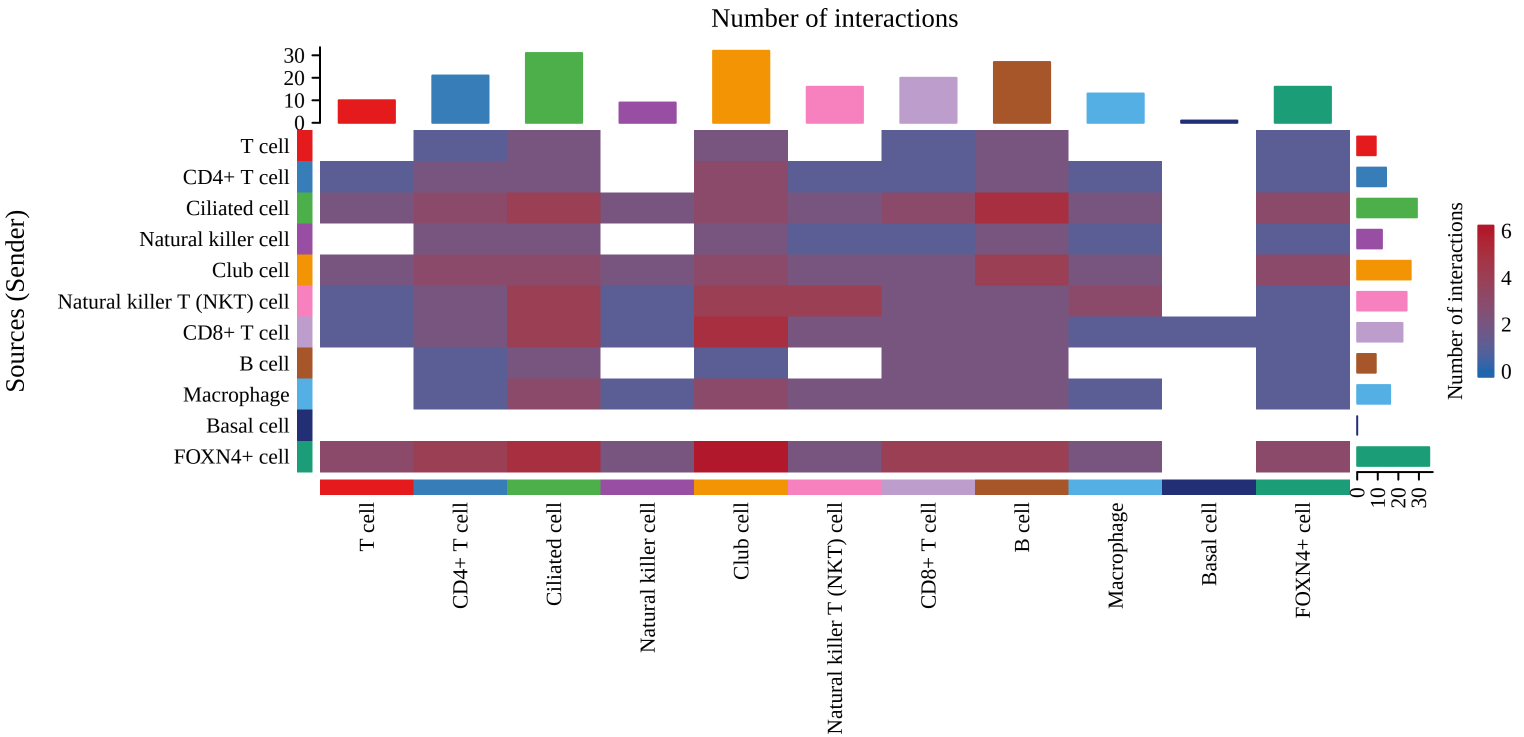

Number of interactions
30
20
10
0
Sources (Sender)
T cell
CD4+ T cell
Ciliated cell
Natural killer cell
Club cell
Natural killer T (NKT) cell
CD8+ T cell
B cell
Macrophage
Basal cell
FOXN4+ cell
Number of interactions
6
4
2
0
